# Progress, Challenges and Prospects of Biomass-Derived Lightweight Carbon-Based Microwave-Absorbing Materials

**DOI:** 10.3390/nano15070553

**Published:** 2025-04-04

**Authors:** Xujing Ren, Meirong Zhen, Fuliang Meng, Xianfeng Meng, Maiyong Zhu

**Affiliations:** 1 School of Materials Science and Engineering, Jiangsu University, Zhenjiang 212013, China; 3220708112@stmail.ujs.edu.cn; 2 School of Management, Jiangsu University, Zhenjiang 212013, China; zmr4233868@ujs.edu.cn; 3 Hangmo New Materials Group Co., Ltd., Huzhou 313310, China

**Keywords:** biomass material, carbon-based composites, microwave absorption

## Abstract

The widespread use of electronic devices in daily life, industry and military has led to a large amount of electromagnetic pollution, which has become an increasingly serious security issue. To eliminate or mitigate such risks and hazards, various advanced microwave absorption technologies and materials have been reported. As a new type of microwave absorber, biomass-derived carbon-based materials have received extensive attention. They have the characteristics of low cost, easy preparation, high porosity and environmental friendliness while retaining the advantageous adjustable dielectric properties, high conductivity and good stability of traditional carbon materials. The development of biomass microwave-absorbing materials not only provides a new idea for solving electromagnetic radiation but also helps to create an environmentally friendly and harmonious environment. Herein, various biomass-derived carbon-based microwave-absorbing materials (MAMs) including plant shells, plant fibers and other potential biomass materials are generalized and discussed including their preparation technology, microstructure design and so on. The two critical factors affecting microwave absorption properties, impedance matching and attenuation characteristics, are analyzed in detail. Finally, the confronting challenges and future development prospects of biomass-based microwave-absorbing materials are pointed out.

## 1. Introduction

Electronic devices that rely on electromagnetic waves (EMWs) have been widely used in various fields such as military [1,2,3], industry [4,5,6], agriculture [7,8,9], food engineering [10,11,12], bioengineering [13,14,15], etc. However, with the development of electronic science and information technology, the ensuing electromagnetic pollution poses a great threat to human health and the environment [16,17,18]. Not only that, electromagnetic radiation also affects human development, leading to cancer cell production, visual impairment, leukemia and other issues, affecting the body′s immunity and DNA system [19,20]. Meanwhile, in the military, EMWs will interfere with weapon systems, such as inaccurate information, failure to detect enemy intrusion targets, and so on. The development of new EMW-absorbing materials is very beneficial to solve these problems and has attracted significant attention [21]. Although traditional MAMs such as ferrite [22,23], conductive polymers [24,25], and metals [26,27,28] have strong microwave-absorbing properties, they have the disadvantages of difficult processing, easy agglomeration, poor stability, large weight and poor impedance matching. Therefore, it is of great significance to develop high-performance MAMs that are thin and lightweight as well as have a wide absorption band, strong absorption capacity and good environmental adaptability [29].

Recently, the biomass-derived carbon-based MAMs have caught the attention of researchers in the field of EMWs absorption due to their low cost, easy availability and potential EMW absorption characteristics. Biomass materials refer to organic materials obtained from different natural substances including animals, plants, wastes and organic products [30,31,32]. They are widely used materials, from energy substances to excellent MAMs. Carbon materials derived from biomass materials have many advantages: abundant reserves, renewable, low cost, good electrical conductivity and high specific surface area; thus, biomass carbon materials have been widely used in capacitors [33,34], ion batteries [35,36], sensors [37,38], electrocatalysts [39,40], and other fields. Particularly, biomass carbon materials possess unique microstructures and a large number of microscopic pores inside the material [41,42]. These pores significantly improve the energy dissipation efficiency of EMWs inside the material. Moreover, they can not only effectively reduce the density and conductivity of the material but also help to improve the impedance matching of the material in the application of EMWs loss, so that the EMWs can enter the material more deeply, resulting in multiple scattering and reflection [43].

**Figure 1:**
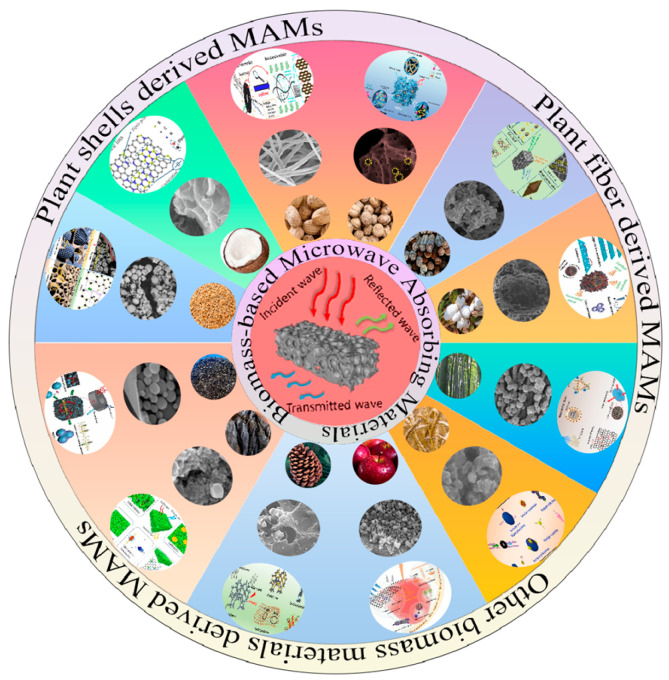
The various lightweight and high performance biomass carbon-based microwave-absorbing materials.

Over the last few years, various light and high-performance biomass carbon-based-MAMs have been reported, as shown in Figure 1. Recently, Li et al. summarized the sources of various biomass materials that can be used for microwave absorption, described the relevant degradation methods and conditions, and summarized biomass-based microwave absorption composites with different attenuation modes and mechanisms [44]. Peymanfar et al. discussed the effect of the morphology of the prepared conjugated carbonaceous structure on the conductivity and polarization loss of the prepared microwave absorber as well as the effect of heteroatoms and doping on the microwave properties of the material [45]. Zhao et al. summarized the research results of carbon-based magnetic fibers in recent years, focusing on the preparation methods, structural differences, microwave absorption properties and structural advantages of carbon-based magnetic fibers [46]. Shi et al. summarized the microstructure characteristics and control mechanisms of EMW absorption in various carbon sphere-based composites, taking into account factors such as composition, morphology, size, and structure [47]. According to the regularity of the structure of biomass carbon materials, Guo et al. described and classified biomass carbon materials with excellent microwave absorption properties [48]. However, there is not yet a comprehensive review focusing on different types of biomass materials for EMW absorption applications. Therefore, in this review, the latest research progress including pure biomass carbon materials and biomass carbon-based composites is summarized. The design strategies and preparation technology are compared and analyzed. Finally, the current challenges and future development prospects of biomass carbon-based MAMs are summarized.

## 2. MA Absorption Mechanisms

When EMWs are incident on the surface of absorbing materials, the interaction process includes the reflection of surface, the multiple reflection contact interface, absorption and transmission inside the material [49,50]. In order to maximize the absorption of EMWs and dissipate them almost without surface reflection and transmission, the ideal absorbing material must have two pivotal characteristics: ① good impedance matching; that is, the impedance gap between the material boundary and the air is required to be as small as possible, and the reflection loss is minimized or even close to 0, so that the EMWs can enter the material to the maximum extent, and ② excellent attenuation capacity; that is, after the EMWs enter the interior of the material, the EMWs can be converted into heat energy or other forms of energy as much as possible to achieve the maximum loss, avoiding re-reflection and transmission [30,51].

**Figure 2:**
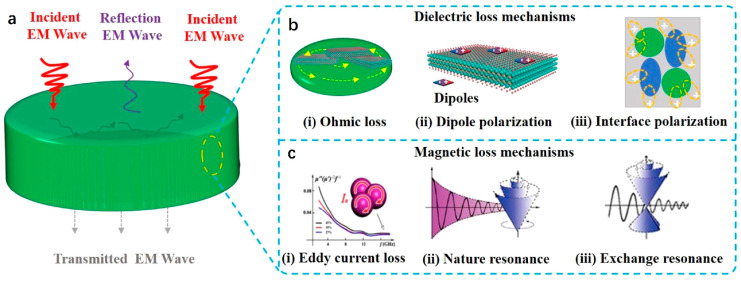
(**a**) Schematic diagram of EMWs absorption mechanism; (**b**) the dielectric loss mechanism; (**c**) the magnetic loss mechanism. Reprinted with permission from Ref. [52] Copyright (2023), Elsevier.

The schematic diagram in Figure 2a shows the interaction process. When the EMWs propagate in space and reach the surface of the EMW-absorbing material. Based on transmission line theory, the reflection of the material surface leads to ongoing reflection, absorption and transmission inside the material. The researchers hope that EMWs can be absorbed to the maximum extent and dissipated almost without reflection and transmission in order to achieve the ideal EMWs absorption, which depends on the impedance matching between EMWs and absorbing materials in free space. However, when the EMWs enter the interior of the material, their energy is attenuated or even dissipated completely, which is one of the qualities of an excellent EMWs absorber. Dielectric loss and magnetic loss are the main mechanisms for materials to absorb EMWs. Dielectric loss includes ohmic loss and polarization loss. Among them, dipole polarization and interface polarization are the attenuation mechanisms of most biomass-based absorbing materials (Figure 2b). At the same time, in the field of microwave absorption, the magnetic loss mechanism mainly includes natural resonance, exchange resonance and eddy current loss (Figure 2c) [52].

### 2.1. Impedance Matching

Impedance matching is a parameter to study the interaction between EMWs and materials as well as the effect of wave transmission in different frequencies [53]. When the EMWs reach the surface of the material through air propagation, most of them will be reflected if the impedance of material surface is quite different from that of the free space (or air). Therefore, meeting the impedance matching between the absorbing material and the free space is the key to realize EMWs’ effective absorption. The reflection coefficient (R) of single-layer absorbing materials meets Formulas (1) and (2) [54,55,56]:

(1) $$R=\left|\frac{Z_{in}-Z_{0}}{Z_{in}+Z_{0}}\right|=\left|\frac{Z-1}{Z+1}\right|$$

(2) $$Z_{in}=Z_{0}{\left(\frac{\mu_{r}}{\varepsilon_{r}}\right)}^{\frac{1}{2}}\tan h\left[j\left(\frac{2\pi fd}{c}\right){\left(\mu_{r}\varepsilon_{r}\right)}^{\frac{1}{2}}\right]$$

Among them, *Z_in_* represents the impedance of the EMWs inside the material, *Z_0_* represents the impedance of the EMWs in free space, *Z = Z_in_*/*Z*_0_ is the transmission impedance at the interface, *f* represents the frequency of the EMWs; *d* is the thickness of the sample, and c is the speed of the EMWs [57]. *μ_r_* represents the complex permeability of the material, and *ε_r_* represents the complex dielectric constant of the material, which are expressed by Formula (3) and Formula (4) [58]:

(3) $$\mu_{r}=\mu^{\prime }-j\mu^{\prime \prime }$$

(4) $$\varepsilon_{r}=\varepsilon^{\prime }-j\varepsilon^{\prime \prime }$$

where *μ*′ is the real part of permeability, *ε*′ is the real part of the dielectric constant, *μ*″ is the imaginary part of permeability, *ε*″ is the imaginary part of the dielectric constant. Meanwhile, *μ*′ and *ε*′ generally represent the maximum storage capacity magnetic and charge energy, while *μ*″ and *ε*″ represent the dissipation ability for magnetic and charge energy [59,60].

The maximum absorption capacity of microwave-absorbing materials can be revealed by the reflection loss (*RL*) of single-layer materials calculated by Formula (5) [61].

(5) $$RL=20\mathrm{lg}\left|\frac{Z_{in}-Z_{0}}{Z_{in}+Z_{0}}\right|=20\mathrm{lg}\left|\frac{Z-1}{Z+1}\right|$$

From Formula (5), when *Z_in_* = *Z*_0_, that is, the impedance of the absorbing material and free space is equal, then the reflection loss is 0, and the material achieves the ideal state of absorbing performance [62]. But in fact, there are no materials with exactly matched impedance. In the design of materials, the impedance of the absorbing material can only be as close as possible to that of free space so that the reflection loss is minimized. Therefore, the reflection loss is an important parameter to evaluate the absorbing performance of the material. When *RL* < 0, it means that the material has a preliminary absorption capacity for EMWs. The greater the absolute value of *RL*, the stronger the absorption capacity. When *RL* < −10 dB, it means that 90% of the EMWs energy is dissipated. When *RL* < −20 dB, it means that 99% of the EMWs energy is dissipated [63]. The growth trend of electromagnetic wave absorption capacity is not obvious after *RL* < −10 dB; therefore, the frequency range exceeding −10 dB is called the effective absorption band.

### 2.2. Attenuation Characteristics

The EMWs generally derive from electric fields and magnetic field radiation. After the EMWs enter the material, the main loss forms include dielectric loss and magnetic loss [64]. Absorption efficiency refers to the conversion of electromagnetic energy into heat energy or other forms of energy. Among them, the electromagnetic wave attenuation ability of MAMs is the key factor. It is worth noting that the electromagnetic dissipation factor (α) is expressed as follows, which can be used to evaluate the dielectric loss and magnetic loss capacity of absorbing materials [65].

(6) $$\alpha =\frac{\sqrt{2}\pi f}{c}\times \sqrt{\left(\mu_{r}^{\prime \prime }\varepsilon_{r}^{\prime \prime }-\mu_{r}^{\prime }\varepsilon_{r}^{\prime }\right)+\sqrt{{\left(\mu_{r}^{\prime \prime }\varepsilon_{r}^{\prime \prime }-\mu_{r}^{\prime }\varepsilon_{r}^{\prime }\right)}^{2}+{\left(\mu_{r}^{\prime \prime }\varepsilon_{r}^{\prime }+\mu_{r}^{\prime }\varepsilon_{r}^{\prime \prime }\right)}^{2}}}$$

It can be seen from Formula (6) that the increase in the imaginary part of the dielectric constant and permeability can enhance the electromagnetic wave attenuation ability of the material [66].

#### 2.2.1. Dielectric Loss

Dielectric loss mainly consumes electromagnetic wave energy by the characteristic electron interaction between the absorbing material and the electric field [52]. Ohmic loss refers to the energy loss caused by the presence of resistance when the current passes through a conductor or medium. In some conductive materials (such as metals), under the action of electric field, energy loss will be generated due to the internal resistance of the material. The polarization loss is mainly caused by ion polarization, electron polarization, dipole polarization and interface polarization [67,68,69]. A microwave is an EMW in the frequency range of 300 MHz–300 GHz; the ion polarization and electron polarization mainly occur in the high-frequency electromagnetic fields of 10^3^ to 10^6^ GHz. So, in most cases, the two polarization effects can be ignored [70,71]. Dipole polarization refers to the process of forming microscopic electric dipoles by the change in charge distribution in molecules or atoms [72]. It occurs at microwave frequencies. Due to the different charge distributions of non-polar molecules and polar molecules, the polarization mechanism is divided into two types. For non-polar molecules, the electron cloud and the nucleus shift under applied electric field. This will form a short dipole moment, resulting in molecular polarization. For polar molecules, the molecules with permanent electric dipole moments are rearranged under the applied electric field, and the dipole moment is oriented along the direction of the external electric field. Therefore, it is also called directional polarization [31,73]. The repeated redirection of the dipole effectively consumes the energy of the EMWs. Interface polarization is a polarization phenomenon formed on the interface of inhomogeneous media. Since the materials on both sides of the interface may have different polarity or conductivity, the electrons or ions will accumulate at the interface under the external electric field and cause the electric dipole moment. The polarization energy is stored in the medium. The rearrangement of charges consumes energy, which in turn leads to the attenuation and loss of electromagnetic energy during the conversion process. Interface polarization can also occur in the microwave region, material defects and grain boundaries [74]. Therefore, dipole polarization and interface polarization are the main loss mechanisms of dielectric loss.

#### 2.2.2. Magnetic Loss

Magnetic loss is related to the energy dissipation of electromagnetic energy. It is mainly caused by the conversion of electromagnetic waves into heat or other forms of energy [75]. Under an external magnetic field, the energy of EMWs is irreversibly converted into heat energy during the process of magnetization and remagnetization. The main loss mechanisms include domain wall resonance, hysteresis loss, natural resonance, exchange resonance and eddy current loss [76]. In these mechanisms, domain wall resonance generally occurs in the low frequency range [77]. Hysteresis loss needs to consume the EMWs energy by overcoming the coercivity in the magnetization process. Importantly, it mainly occurs in strong electromagnetic fields. Therefore, the main factors causing magnetic loss are natural resonance, exchange resonance and eddy current loss. The natural resonance is related to the anisotropy of magnetic particles. The degree of anisotropy will affect the absorption energy of the EMWs. It is worth noting that smaller magnetic particles have stronger anisotropy [78,79]. Therefore, the absorption properties of the material can be adjusted by controlling the anisotropy of the magnetic particles. Exchange resonance occurs at higher frequencies. It is related to the energy exchange between the particles and surface anisotropy. The eddy current refers to the closed current loop inside the ferromagnetic material due to the external alternating magnetic field or electric field [80]. The generation of an eddy current will lead to the conversion of the EMWs energy into heat to form an eddy current loss. Eddy current loss can be expressed as shown below [81]:

(7) $$C_{0}=\mu^{\prime \prime }{\left(\mu^{\prime }\right)}^{-2}f^{-1}=\frac{2}{3}\pi \mu_{0}{\mathrm{d}}^{2}\sigma$$

Among them, *C_0_* is the eddy current loss coefficient, *μ_0_* is the vacuum permeability, *d* is the thickness of the sample, and σ is the conductivity of the material. The eddy current loss is the main influence mechanism of magnetic loss when *C_0_* does not change with frequency or its fluctuations are very small. On the contrary, when *C_0_* changes and fluctuates greatly with the frequency, its influence can be ignored. The natural resonance and exchange resonance play a major role in magnetic loss. In addition, it can be seen from Formula (7) that the *C_0_* value is related to the material size. When the material thickness exceeds the threshold value, the skin effect will be produced and lead to impedance mismatch, thus decreasing the microwave absorption performance [82,83].

## 3. Biomass-Derived Microwave Absorption Materials

Carbonization refers to the process in which biomass is decomposed into highly graphitized carbon- and nitrogen-rich porous carbon materials by thermochemical treatment under air-insulated conditions. The pores in the biomass carbon endow the material with good interfacial polarization for the absorption mechanism, increasing the proportion of the air medium and improving the impedance matching to a certain extent. In addition, compared with traditional absorbing materials, biomass materials have many advantages, such as abundant pores, excellent conductivity, good thermal stability, strong environmental suitability, and wide sources. Therefore, biomass-derived MAMs provide a promising design strategy and pathway for developing lightweight and high performance MAMs [84].

### 3.1. Plant Shells-Derived MAMs

Driven by the dual needs of electromagnetic pollution control and functional material innovation, agricultural waste has become a key research object for researchers in the field of microwave absorption. Natural shell resources such as rice husk, coconut shells, peanut shells and walnut shells showed excellent dielectric loss characteristics and EMWs absorption potential due to their unique multi-level pore structure, carbon-based skeleton and rich silicate components. This kind of biomass-based materials not only effectively solved the technical bottlenecks related to the high cost, high density and heavy environmental load of traditional absorbers it also achieved broadband and strong attenuation absorbing efficiency through component regulation and structural design. It provided a green solution for the development of a lightweight and sustainable new generation of absorbing composites, and it showed broad application prospects in the fields of 5G communication, stealth technology and electromagnetic protection.

#### 3.1.1. Rice Husk as Precursor

Rice husk is a common crop by-product, which is rich in silicon and carbon [85]. The porous structure of natural rice husk endows rice husk-derived carbon with excellent dielectric loss properties. At the same time, the large specific surface area of rice husk-derived carbon is conducive to promoting the effective scattering of EMWs. These unique physical properties cooperate with each other, making the MAMs with rice husk-derived carbon as the precursor able to achieve a significant attenuation of EMWs. In addition, the surface of rice husk-derived carbon is rich in functional groups, which can trigger dipole polarization relaxation and further promote the attenuation of electromagnetic energy [86].

**Figure 3:**
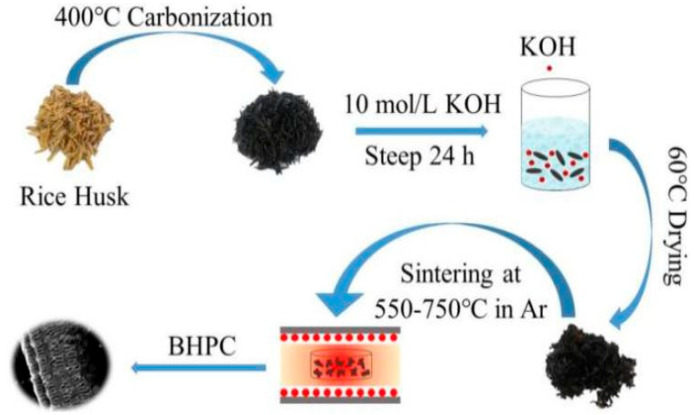
Schematic diagram of rice husk based multi-pore absorbing material synthesis. Reprinted with permission from Ref. [87] Copyright (2022), Elsevier.

Simple carbonization and activation treatment is a common processing technology. Wu et al. prepared porous rice husk-derived carbon materials using rice husk as raw material by KOH activation and one-step carbonization. The schematic diagram of the synthesis process is shown in Figure 3. It was found that the minimum reflection loss (*RL_min_*) was −47.463 dB and the effective absorption bandwidth (*EAB*) was 3.402 GHz when the sample thickness was 2.8 mm and the heat treatment temperature was 650 °C. By controlling the heat treatment temperature, the pore structure of the sample was adjusted, and the dielectric constant and impedance matching were improved. The pore structure (micropore, mesopore, or macropore) was a major factor for the enhanced microwave absorption performance. The small pores formed defects on the material surface and acted as polarization centers, resulting in dipole polarization. The mesopores increased the specific surface area, forming many solid space interfaces and leading to interfacial polarization; the large pores formed a conductive network, which enhanced the conductive loss. Therefore, rice husk-derived carbon materials have broad prospects as MAMs with strong absorption performance and lightweight [87].

Although pure biomass-derived carbon materials have good dielectric loss ability, a single electric loss mechanism will lead to the impedance mismatch and decrease the attenuation of EMWs. In view of this, many researchers have chosen to combine them with magnetic materials (such as metal particles, metal oxide, etc.) to enhance the magnetic loss ability of MAMs. Wu et al. used rice husk as a raw material; after it was carbonized and activated, nickel particles were coated on the surface of the rice husk-derived carbon to obtain the Ni/C composites by electroless plating. The introduction of nickel particles endowed the Ni/C composites with enhanced saturation magnetization and improved the magnetic loss capacity. The porous structure and interconnected pores constituted a unique three-dimensional network structure, which made a large number of EMWs reflect inside the material, prolonged the transmission path of EMWs and led to energy dissipation. Using 0.2 mol/L NiSO_4_ for chemical plating and a sample thickness of 2.7 mm, the *RL_min_* of the sample was −58.50 dB and the *EAB* reached 3.51 GHz [88]. Liang et al. used different moles of Ni(CH_3_COO)_2_·4H_2_O and Co(CH_3_COO)_2_·4H_2_O to impregnate the natural rice husk and then a simple annealing treatment was performed to prepare NiCo_2_/C MAMs. Its *RL_min_* is −55.62 dB, and the *EAB* was 3.6 GHz with 30 wt% of filler load and 3.57 mm of sample thickness [89]. Different from Liang et al., Li et al. firstly prepared porous carbon with a large specific surface area by calcination and potassium hydroxide activation, and then they introduced Fe-Co alloy particles to obtain nano-MAMs. The results indicated under a thinner sample thickness (1.44 mm) that the *RL_min_* was better, with a value of −68.11 dB, and the *EAB* was higher, with a value of 3.76 GHz. The reason can be explained that the porous carbon originated from rice husk calcination had a lotus root-like structure, which helped with loading of magnetic particles [90].

**Figure 4:**
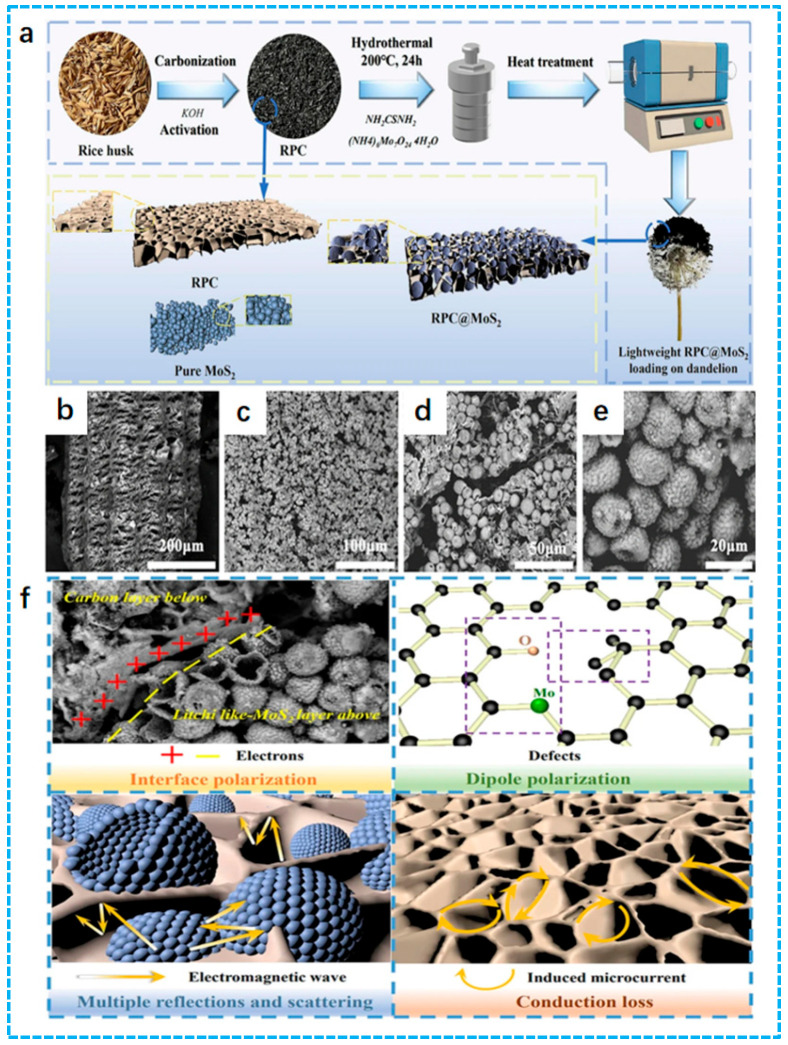
(**a**) Schematic diagram of the synthesis process of RPC@MoS_2_ composite; SEM image of materials (**b**) RPC; (**c**) pure MoS_2_; (**d**,**e**) RPC@MoS_2_-600; (**f**) The possible EMA mechanism of RPC@MoS_2_-600. Reprinted with permission from Ref. [91] Copyright (2023), Springer.

Abundant pores of porous carbon not only provide a pathway for the incidence of EMWs but also provide more opportunities for magnetic particles entering the material by co-deposition, which can trigger the magnetic resonance effect and effectively enhance the impedance-matching performance of the material. Yao et al. prepared a lightweight rice husk-derived porous carbon (RPC)@MoS_2_ composites with a lychee-like porous structure using rice husk as a precursor. The synthesis diagram is shown in Figure 4a. After high-temperature carbonization and hydrothermal reaction, the sample displayed an obvious porous structure (Figure 4b–e). When the sample thickness was 2.0 mm and the temperature was 600 °C, *RL_min_* and *EAB* reached −50.69 dB at 12.73 GHz and 6.375 GHz, covering the entire Ku band. The EMWs absorption mechanism is shown in Figure 4f; the three-dimensional carbon skeleton derived from rice husk creates favorable conditions for the effective adhesion of MoS_2_ due to its high specific surface area. MoS_2_ was uniformly grown in the porous carbon framework and pores derived from rice husk with a unique lychee-like morphology, which improved the absorption performance and enhanced its attenuation effect on EMWs [91].

**Figure 5:**
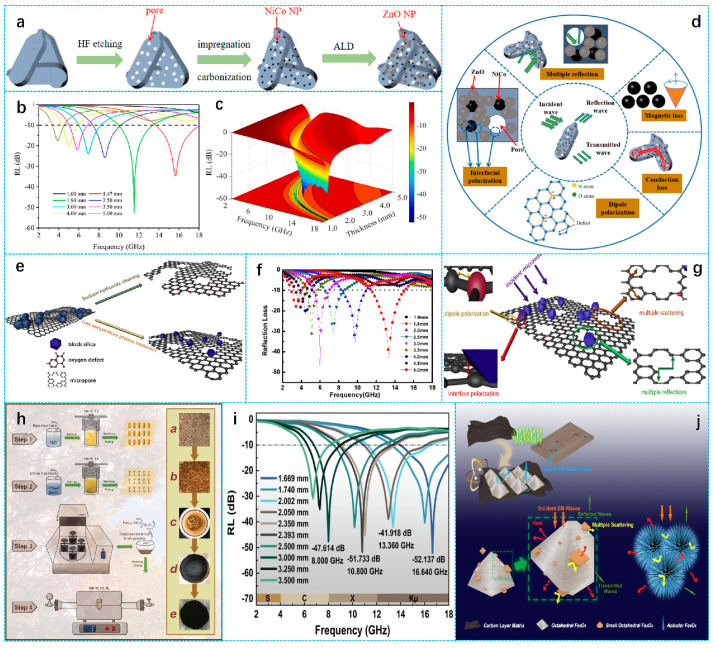
(**a**) The preparation flowchart of ZnO/NiCo/C composites; (**b**,**c**) the RL curves of different thicknesses and the three-dimensional representation of the corresponding RL values; (**d**) the schematic diagram of microwave absorption mechanism. Reprinted with permission from Ref. [92] Copyright (2024), Elsevier. (**e**) Synthetic processing renderings of RHC; (**f**) RL 2D image of PRHC; (**g**) microwave absorption mechanism diagram of PRHC. Reprinted with permission from Ref. [93] Copyright (2020), Elsevier. (**h**) The magnetization, carbonization and activation steps of Fe_3_O_4_/C composites: (a) Hydrothermal pretreatment, (b) Desiliconization pretreatment, (c) Ball mill processing, (d) Metal salt and activator pretreatment, (e) N_2_ Atmosphere calcination; (**i**) the reflection loss curve; (**j**) the schematic diagram of EMWs absorption mechanism. Reprinted with permission from Ref. [94] Copyright (2021), Elsevier.

The design strategy and preparation technology have a decisive impact on microstructures and their components, which will affect the electromagnetic loss mechanism. Yan et al. prepared ZnO/NiCo/C MAMs by annealing and atomic layer deposition, as shown in Figure 5a. In the strategy, the electromagnetic parameters were precisely controlled by adjusting the deposition thickness. Although the nickel and cobalt alloy can enhance the magnetic loss, due to the impedance mismatch between excessive metal particles and carbon materials, the MA properties of NiCo/C composites were unsatisfactory. In the study, the ZnO with lower dielectric constant was applied to tune the electromagnetic properties, high temperature resistance and oxidation resistance of the materials. When the thickness of the ZnO/NiCo/C sample was 1.42 mm, the *RL_min_* and *EAB* were −52.5 dB and 4.48 GHz (Figure 5b,c), and the corresponding absorption mechanism is shown in Figure 5d. Through multi-mechanism synergy, the material achieves excellent microwave absorption performance: The porous structure optimized impedance matching and promoted EMW penetration and multiple scattering. The O and N atom defects and the C=O functional group acted as dipole centers, triggering strong dipole polarization. ZnO and NiCo nanoparticles formed a heterogeneous interface with the carbon matrix to enhance the interfacial polarization. The graphite carbon network was intertwined with the magnetic alloy to construct a conductive path and strengthen the conductive loss. NiCo nanoparticles broaden the magnetic loss band through natural resonance and exchange resonance [92]. Li et al. prepared silica hybrid porous carbon materials derived from rice husk by combining pyrolysis and plasma processing methods. The treatment effect of rice husk-derived carbon (RHC) during the synthesis process is illustrated in Figure 5e. On this basis, PRHC was obtained by the further treatment of plasma engineering for half an hour. The *RL_min_* of the prepared absorbing material was −43.0 dB at a thickness of 1.5 mm (Figure 5f) and a temperature of 900 °C; the *EAB* reached 3.68 GHz. From the absorption mechanism of Figure 5g, the optimized performance was mainly due to the structural defects caused by oxygen plasma technology and the retention of silicon hybridization from the rice husk [93]. These characteristics enhanced the Debye relaxation, dipole polarization, and interfacial polarization closely related to the enlarged surface area, thereby enhancing the microwave absorption performance. Shu et al. prepared needle-like and octahedral Fe_3_O_4_/C composites using an improved ball-milling process and different doses of activators and magnetizing agents. Through the improved ball-milling process, the obtained rice husk powder was more uniform, resulting in a more thorough reaction. The synthesis diagram of Fe_3_O_4_/C composites is shown in Figure 5h. When the material thickness was 1.669 mm, the *RL_min_* was −52.14 dB (Figure 5i), and the *EAB* was about 13 GHz, which nearly covers all the C, X and Ku bands, showing broadband absorption characteristics. The EMWs absorption mechanism is shown in Figure 5j. The composite material had a compact needle-like morphology and an octahedral structure with multiple regular corners, which effectively enhanced the multiple scattering of the EMWs. The addition of a Fe_3_O_4_ magnetic material brought different interfacial polarization. At the same time, it also led to the emergence of natural resonance and exchange resonance. The dielectric loss and magnetic loss were effectively enhanced [94].

Singh et al. [95] used banana stem (BAN) and rice husk powder (RH) as carbon sources to prepare a BAN/RH/epoxy resin polymer composite with a porous skeleton structure and good absorbing effect by sodium hydroxide, ultrasonic treatment and artificial stratification. The researchers used ultrasonic alkalization to modify the original fibers to enhance compatibility, removed impurities and increased porosity, further forming a composite with a unique structure. When the thickness was 3 mm, the sample showed −14.01 dB of the *RL_min_* and 2.13 GHz of *EAB*. This modification method endowed the composite material with new characteristics, which made it have wide application potential in the design of stealth and coating materials for various electronic devices. Recently, a new multifunctional foam concreted by rice husk ash replacing sulfoaluminate cement was reported by Bai et al. [96]. The researchers have comprehensively applied various means such as alkali activation technology and additives to optimize the performance of composite materials, diversified their functions and enhanced their environmental friendliness. After adding 1 wt% rice husk ash, the *RL_min_* and *EAB* of the sample with 9 mm thickness reached −19.7 dB and 2 GHz, respectively. The compressive strength and thermal insulation effect of the material were improved by alkali activation technology. It provided a new idea for the development of new multifunctional building materials with energy saving and environmental protection characteristics, and it also provided a new way to deal with waste rice husks. Both of them were treated with NaOH activation. The selection of NaOH was based on its unique physicochemical properties, which can promote the expansion of the fiber, increase the specific surface area and form a porous structure, thereby improving the penetration efficiency of the surfactant. In addition, the NaOH-induced mercerization treatment allowed the fiber surface to appear glossy, adjusted the reflective properties of the material, and enhanced the absorption efficiency of EMWs or chemicals. At the same time, the alkaline treatment process effectively improves the tensile strength and wear resistance of the fiber by increasing the degree of cellulose crystallization.

As a sustainable material, rice husk has a natural plant fiber structure and can become biomass carbon with a porous structure under high-temperature carbonization. After compounding with magnetic mediums (such as metal, alloy, ferrite etc.), they endowed the biomass carbon magnetic loss mechanism. The obtained composites were a promising material to solve electromagnetic pollution. The adjustment of the porous structure of rice husk carbon through different techniques can enhance the secondary and even multiple reflection of materials to EMWs. In addition, the use of rice husk instead of other costly materials can not only save costs but also provide new solutions for the sustainable development of the environment.

#### 3.1.2. Coconut Shell as Precursor

Coconut shell is a kind of lignocellulosic biomass, which has a multi-layer porous structure and can transport water and nutrients [97]. Its unique natural structure is more conducive to the formation of porous carbon, which is helpful for the reflection and scattering of EMWs. During carbonization, within a certain range, the specific surface area and pore volume of coconut shell-derived carbon will gradually increase with the increase in carbonization temperature, showing a multi-stage porous structure, which enhances the wave absorption performance of MAMs [98,99,100].

**Figure 6:**
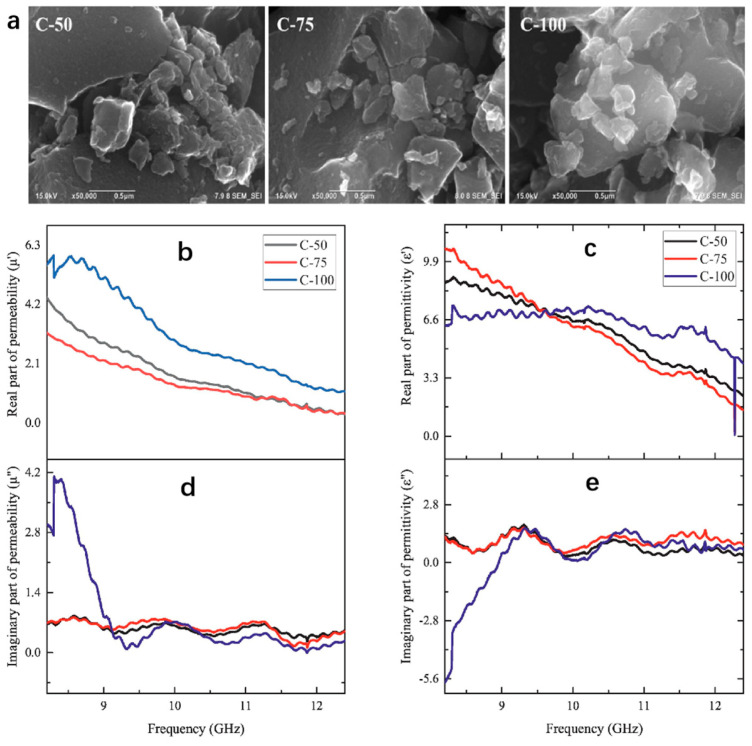
(**a**) The morphological characteristics of activated carbon subjected to varying grinding durations, and (**b**–**e**) shows the relative complex permeability and permittivity of C-50, C-75, and C-100 samples. Reprinted with permission from Ref. [105] Copyright (2022), Elsevier.

Usually, coconut shells release a large amount of gas during carbonization and activation, resulting in microspores and channels. This will help to improve the electromagnetic properties of the material [98]. In addition, coir fiber contains about 38% to 50% carbon [101]. As a key component of microwave energy absorption, carbon can effectively convert microwave energy into heat [102]. Therefore, coir fiber has the potential to become one of the alternative materials for microwave absorption. Wang et al. [103] activated the coconut shell powder with potassium hydroxide as an activator; then, they carbonized them at high temperature and successfully prepared honeycomb-like carbon. By adjusting the carbonization temperature and activator ratio, it was found that when the sample thickness was 1.5 mm, the temperature was 600 °C and the activator was 0.5:1, the *RL_min_* reached −37.23 dB and the *EAB* was 2.88 GHz. Yusuf et al. used a similar method to prepare porous carbon-absorbing materials. Under the carbonization temperature of 750 °C, the *RL_min_* was −45.6 dB, and the *EAB* was 3.59 GHz. The results indicated that the appropriate carbonization and activation technology endowed the carbon with a porous structure, resulting optimal impedance matching [104]. Considering the great application potential of activated carbon, Widanarto et al. used grinding technology to extract some activated carbon from coconut shells to achieve their absorption properties for the X-band. The structure, morphology and microwave-absorbing properties of the prepared fullerene-c70-encapsulated activated carbon were changed by changing the grinding time. The SEM micrographs of the activated carbon crushed at different grinding times are shown in Figure 6a. C-50, C-75 and C-100 represent the micro-grinding morphology of activated carbon at 50, 75 and 100 min, respectively. The formation of carbon particles with interparticle pores provided a large specific surface area, which was suitable for strong interaction with external electromagnetic radiation. It can be seen from Figure 6a that the growth and nucleation of C-100 samples became more prominent due to the prolongation of grinding time. It was the best in structure and morphology, and it had the best EMW absorption performance. The sample exhibits an obvious absorption band near 9 GHz with an *RL_min_* of −23.0 dB and *EAB* of 3 GHz. From Figure 6b,c, it can be seen that compared with C-50 and C-75, the C-100 samples have higher real permeability values and more stable real dielectric constants, indicating that C-100 had higher magnetic and electrical energy storage capacity [105].

However, the pure coconut shell-derived carbons did not exhibit desirable microwave-absorbing properties; the reason was that these researchers only focused on the preparation process and did not consider impedance matching.

**Figure 7:**
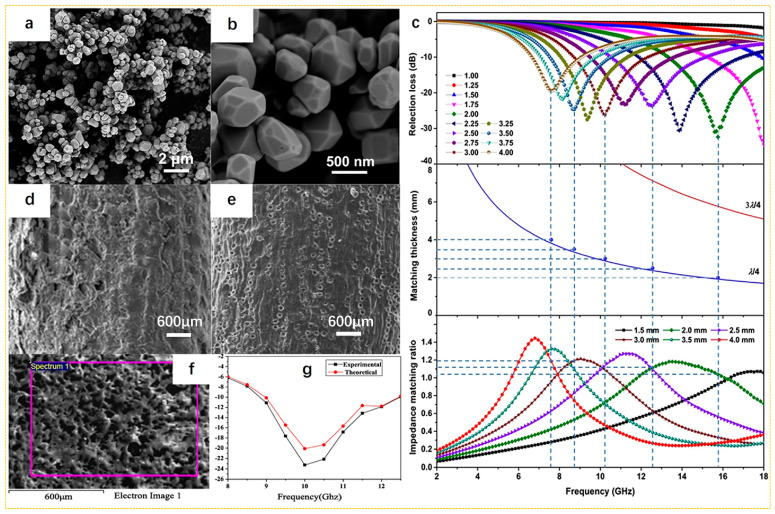
(**a**,**b**) SEM images of pure TiP_2_O_7_ nanoparticles; (**c**) the reflection loss characteristics of TiP_2_O_7_/C composites and the corresponding matching thickness and peak frequency. Reprinted with permission from Ref. [98] Copyright (2020), Elsevier. (**d**–**f**) SEM images of untreated and treated coconut shell fiber samples and coconut shell powder epoxy resin composites; (**g**) reflection loss of composite materials. Reprinted with permission from Ref. [99] Copyright (2020), Springer.

In order to optimize the microwave-absorbing properties, various hybrid materials were developed. Huang et al. [98] successfully prepared TiP_2_O_7_/C composites by one-pot synthesis technology and heat treatment technology using coconut shells as a carbon source. The TiP_2_O_7_ had a unique structure with a three-dimensional framework octahedron of TiO_6_ and a P_2_O_7_ double tetrahedron sharing angle. Form the SEM image of Figure 7a,b, this unique polyanion configuration enabled carriers to move, thereby endowing TiP_2_O_7_ with polaron conductivity and excellent electromagnetic properties [106]. After coupling with carbon, TiP_2_O_7_ with an irregular polyhedral morphology and hundreds of nanometer-sized nanoparticles was wrapped on the surface of a carbon matrix. The obtained TiP_2_O_7_/C composite exhibited excellent microwave absorption performance. At a thickness of 2 mm, the *RL_min_* was −32.4 dB, and the *EAB* reached about 6 GHz (Figure 7c). In view of the perspective of the absorption mechanism, the extensive surface and interface between the TiP_2_O_7_ particles and carbon matrix provided abundant polarization active sites when exposed to external electromagnetic radiation, thereby improving the microwave absorption effect. In addition, the carbon matrix enhanced microwave attenuation due to its significant electrical loss capability and porous structure. Mishra et al. prepared coconut shell/epoxy resin composites by mixing different proportions of coconut shell powder with epoxy resin and curing agents. From the SEM image in Figure 7d–f, after ultrasonic treatment and addition of acetone–methanol mixture, the surface roughness was improved, and the coconut shell powder exhibited good network dispersion in the epoxy resin. When the filling rate was 40 wt%, the *RL_min_* was −23.5 dB at 10 GHz (Figure 7g) [99].

**Figure 8:**
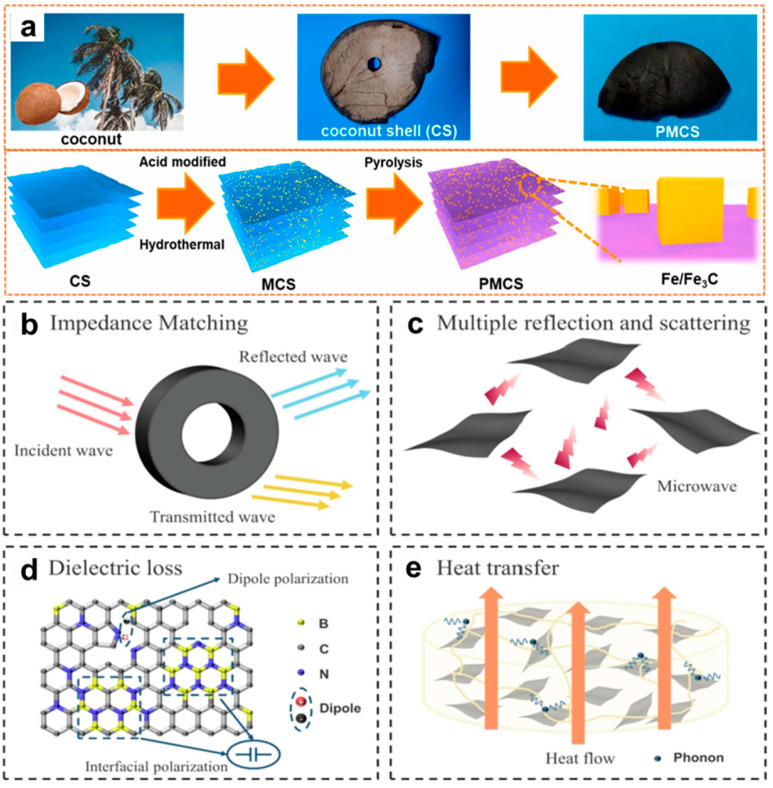
(**a**) A schematic diagram of the process for preparing PMCS. Reprinted with permission from Ref. [107] Copyright (2022), Elsevier. The microwave absorption principle diagram of a BCN nanosheet (**b**–**e**) heat transfer diagram of BCN/NR. Reprinted with permission from Ref. [108] Copyright (2022), Elsevier.

Yang et al. reported on the synthesis of a novel magnetic Fe/Fe_3_C composite combining hydrothermal and carbonization techniques using coconut shells as a raw material. The transformation process from a coconut shell (CS) sheet to a pyrolysis-modified coconut shell (PMCS) is shown in Figure 8a, in which the CS reacted with citric acid and potassium ferricyanide to form the modified coconut shell (MCS), and then the PMCS was obtained by pyrolysis. It was worth noting that during pyrolysis at higher temperature, a large amount of gas was released owing to the internal components decomposing, resulting in the Fe/Fe_3_C nanocubes having a rough and porous surface. This structure can increase the surface area and may enhance the interfacial polarization. By controlling the carbonization temperature, the Fe/Fe_3_C nanocubes’ irregular and porous structure was realized, which improved the interfacial polarization of the composite. An excellent *RL_min_* of −48.87 dB and *EAB* of 7.94 GHz were obtained, indicating that 99.998% of the electromagnetic energy was absorbed [107]. Mou et al. chose coconut shell powder, H_3_BO_3_ and CO(NH_2_)_2_ to make a biomass-derived borocarbonitride (BCN) nanosheet, and then they mixed it with natural rubber to obtain BCN/NR composites. The BCN/NR’s *RL_min_* and *EAB* reached −54.24 dB and 4.16 GHz with 1.4 mm thickness. The BCN material effectively reduces the surface reflection due to its excellent impedance characteristics, enabling most of the microwave energy to be absorbed by the absorber. At the same time, due to the high specific surface area of the BCN material, the EMW generates multi-level reflection and scattering inside (Figure 8c), which enhances the electromagnetic energy loss capability. In addition, the heterogeneous composites composed of C-N, C-B and other defects can be analogized to electric dipoles (Figure 8d), which improves the dielectric relaxation effect. In addition, the heat transfer model of BCN/NR composites is shown in Figure 8e, where BCN is uniformly dispersed in the NR material, which is conducive to the formation of thermally conductive connection channels and networks, thereby promoting the diffusion of phonons in the BCN/NR composites [108].

The above discussions indicated that coconut shell is a resource-rich and renewable biomass material that can obtain a considerable porous structure through simple carbonization. The unique structure characteristics were conducive to the preparation of biomass-derived carbon with abundant functional groups and a large specific surface area, which can hybridize with other materials to form new material systems. At the same time, the high porosity can effectively adjust the dielectric constant and in turn enhance the attenuation of EMWs. In a word, coconut shell-derived carbon can be a new strategy and scheme for the exploitation of low cost, lightweight, high-performance MAMs.

#### 3.1.3. Other Biomass Shells as Precursors

**Figure 9:**
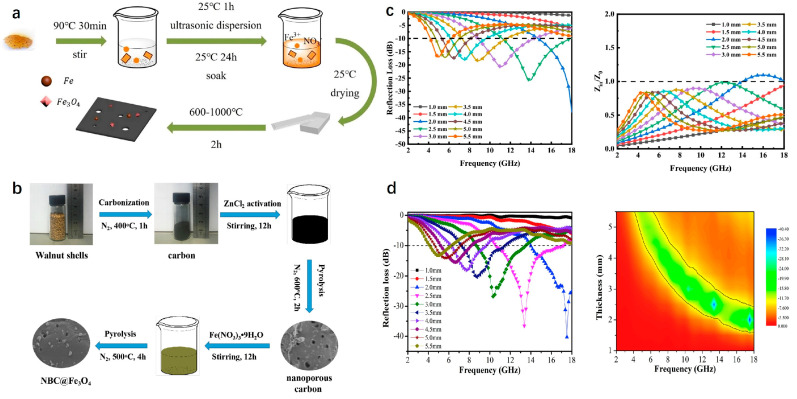
(**a**,**b**) Flowchart of synthesis and preparation of almond shell-based absorbing materials and walnut shell-based absorbing materials; (**c**) reflection loss curve and impedance-matching diagram of almond wood shell-based absorbing materials; (**d**) 2D and 2D contour map of RL values of walnut shell-based absorbing materials. Reprinted with permission from Refs. [112,113] Copyright (2022, 2019), Springer, Elsevier.

With the increasing attention and in-depth research of researchers, besides rice husk and coconut shell, more and more biomass shell-derived carbon MAMs were developed. They included pumpkin seed shells, peanut shells, walnut shells, almond shells, melon seed shells, and so on. Similarly, these materials exhibited a high level of output and were not constrained by concerns regarding the scarcity of primary product resources [109,110]. Due to their cost-effectiveness and porous architecture, they were receiving widespread attention in microwave absorption fields. For example, Zhang et al. used pumpkin seed shells as the matrix; after simple carbonization and activation treatment, the dielectric properties and morphology were optimized by controlling the amount of activator and heating temperature. When the filling rate was only 10 wt%, the *RL_min_* reached −50.55 dB and the *EAB* was 7.4 GHz [111]. Gong et al. prepared the C/Fe_x_O_y_ composites using almond wood shells and ferric nitrate as raw materials under simple electrostatic adsorption and high-temperature calcination. As shown in Figure 9a, the content of iron nitrate and heat treatment temperature were the key factors affecting the microwave-absorbing properties of the sample. With 22.3 wt% of iron nitrate and a temperature of 600 °C or 1000 °C, the *RL_min_* was −37.9 dB and the *EAB* was 7.04 GHz (Figure 9c) [112]. Zhou et al. synthesized the nanoporous biomass carbon@Fe_3_O_4_ (NBC@Fe_3_O_4_) composites by the flow in Figure 9b. Firstly, nanoporous carbon was prepared by walnut shell carbonization, ZnCl_2_ activation and pyrolysis treatment in N_2_ atmosphere. Subsequently, Fe_3_O_4_ nanoparticles were synthesized in situ on the surface of NBC under 500 °C in N_2_ atmosphere. The obtained NBC@Fe_3_O_4_ sample with 0.5 mmol of ferric nitrate exhibited the best microwave-absorbing properties. The *RL_min_* was −40.3 dB at 17.5 GHz and the *EAB* was 6.6 GHz with 2.0 mm thickness (Figure 9d). Although the preparation methods of Gong et al. and Zhou et al. were different, the factors affecting the absorbing properties were basically the same. The multi-stage porous structure of the biomass carbon caused multiple reflections and the scattering of incident EMWs. The existence of Fe_3_O_4_ nanoparticles induced more interface defects, resulting in enhanced interface polarization and the further attenuation of EMWs energy. It should be noted that an appropriate amount of iron nitrate can effectively improve the reflection loss. However, excessive addition will lead to the impedance mismatch and reduce the reflection loss [113].

**Figure 10:**
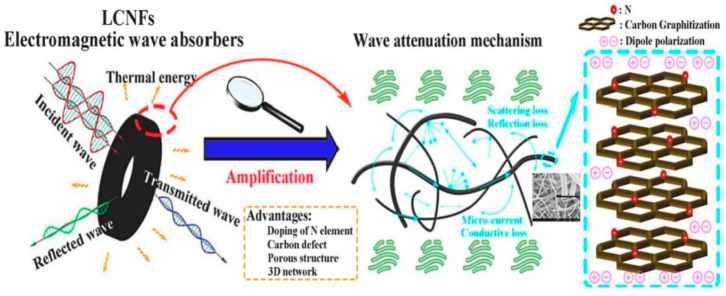
EMWs absorption mechanism diagram of light lignin-based carbon nanofibers. Reprinted with permission from Ref. [115] Copyright (2022), Elsevier.

By combining peanut shell-derived biomass porous carbon (BPC) with conductive polymer polyaniline (PANI), Xu et al. successfully synthesized PANI/BPC composites through carbonization, activation and oxidative polymerization. Here, the coral-like PANI randomly adhered to or acted as a bridge to connect the fracture gullies on the surface of the porous carbon to construct a more complex network structure. The special structure can effectively adjust the dielectric constant and optimize the impedance matching. The *RL_min_* reached −40.89 dB at 2.6 mm and the *EAB* reached 4.24 GHz at 2.1 mm [114]. Du et al. reported on polyacrylonitrile/carbon composites using melon seed shells and peanut shells as carbon sources, respectively. Compared with peanut shells, the composite using melon seed shells as the carbon source exhibited better energy storage and electromagnetic absorption performance. It was attributed to the larger average molecular weight, preferable linear structure and lower polydispersity of melon seed shells, which greatly led to considerable conductive loss. When the lignin content increased by 30 wt%, the melon seed shell-based composite had the best microwave-absorbing performance. At 7.98 GHz and a thickness of 3.0 mm, the *RL_min_* was −37.2 dB. The corresponding absorption mechanism is shown in Figure 10. The doping of the N element in the composite material produced multiple polarizations, thereby enhancing the relaxation loss. At the same time, the high conductivity of the composite material also enhanced the reflection of the incident wave. In addition, amorphous carbon generated during the high-temperature calcination process resulted in the formation of disordered sites on the surface of the material, which led to defect polarization and dipole polarization relaxation, and further caused interface polarization, which helped to improve its EMWs absorption ability [115].

**Figure 11:**
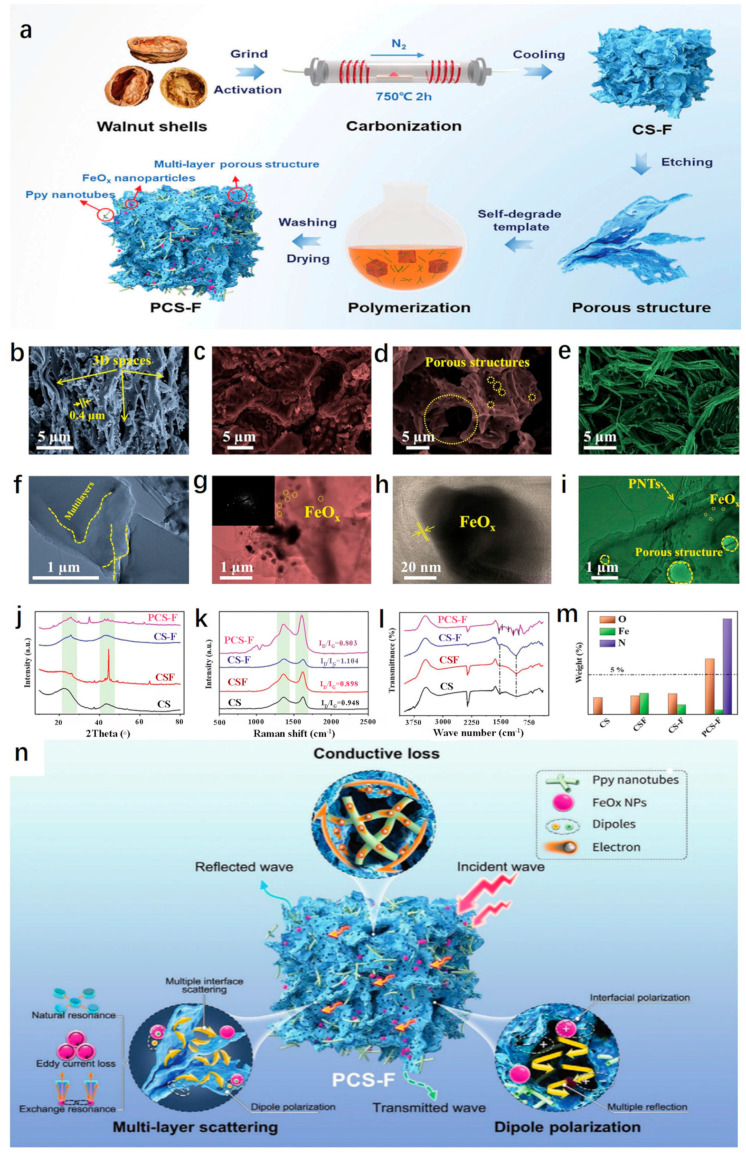
(**a**) Biomimetic composite material synthesis schematic diagram; (**b**–**e**) SEM images of CS, CSF, CS-F and PCS-F; (**f**–**i**) TEM images of CS, CSF and PCS-F; (**j**) XRD diffraction pattern; (**k**) Raman spectrum; (**l**) FT-IR spectra; (**m**) weight ratio of component signal; (**n**) schematic diagram of EMWs absorption mechanism of composite materials. Reprinted with permission from Ref. [116] Copyright (2024), Wiley.

Inspired by the structure of biological nests in nature, Elhassan et al. prepared the carbonized walnut shell (CS) by one-step calcination; FeCl_3_ was used to decorate the CS by the chemical polymerization process, and the porous sample (CS-F) was obtained by etching with hydrochloric acid. After cleaning and drying, a biomimetic composite (PCS-F) with an ant nest structure was developed by polymerizing and coating the conductive polypyrrole nanotubes (PNTs) (Figure 11a). From Figure 11b–i, it can be seen that CS-F exhibited many interesting features compared with CS, including a wide range of 3D networks, bubble-like structures and more abundant micropores. Based on the structure and parameters (Figure 11j–m), excellent microwave-absorbing properties were obtained, and the ultra-thin sample (1.6 mm) displayed excellent *RL_min_* (−67.6 dB) and *EAB* (5.4 GHz) values. From the microwave-absorbing mechanism (Figure 11n), the ant nest structure promoted the frequent multiple reflection and scattering of EMWs in the hollow and multi-layered samples. At the same time, due to the different conductivity, local charge accumulation occurred at the C/PNTs, C/FeO and solid/pore interfaces, which caused interfacial polarization. It was worth noting that the complex 3D structure and gradient pore structure of PCS-F also endowed the material with good thermal insulation performance and hydrophobicity, which provided inspiration and valuable insights for the development of multifunctional EMW-absorbing materials [116]. Similarly, Li et al. prepared a light Fe_3_O_4_@C/C absorber by the pyrolysis-hydrothermal method. Different from the former, the latter mixed polyvinyl alcohol, urea, FeCl_3_·6H_2_O and carbonized walnut shell. After a series of treatments, Fe_3_O_4_@C nanospheres were aggregated and tightly wrapped on the surface of biomass carbon. When the matching thickness was 2.46 mm, the *RL_min_* was −56.61 dB, and the *EAB* reached 5.68 GHz, which covered the whole Ku band [117].

Wang et al. successfully synthesized multidimensional pine nut shell-derived carbon@nickel-cobalt-layered double hydroxide@nickel chain (C@NiCo-LDHs@Ni) aerogel by a simple solvothermal and freeze-drying method. The C@NiCo-LDHs@Ni aerogel has a multi-dimensional structure composed of 1D Ni chains, 2D NiCo-LDHs and a 3D carbon skeleton. The 3D structure provided favorable conditions for the growth of 2D NiCo-LDHs. The 2D layered NiCo-LDHs were uniformly dispersed on the surface of biomass-derived carbon and wrapped on the carbon skeleton, which effectively filled the defects. It avoided the skin effect caused by alternating electromagnetic fields and also provided the possibility for the combination of Ni chains. A large number of functional groups and holes on the surface of the carbon skeleton and 2D layered NiCo-LDHs enhanced the dipole polarization. The eddy current loss, exchange resonance and natural resonance caused by the 1D Ni chain enhanced the magnetic loss. In addition, C@NiCo-LDHs@Ni aerogels also had good thermal insulation, compression resistance and corrosion resistance. So, the reasonable component design, dimensional gradient structure and magnetic loss endowed the C@NiCo-LDHs@Ni aerogel with an excellent microwave-absorbing performance. When the filler ratio was 30 wt% and the matching thickness was 2.5 mm, the *RL_min_* reached −57.4 dB and the *EAB* was 6.4 GHz [118].

Liu et al. designed a Ni/PDCs/biomass ceramic composite by loading magnetic metals into peanut shells through a polymer-derived ceramic process and suspension impregnation method. The synergistic effect of Ni/PDCs significantly enhanced the impedance matching. The BET results showed that the synthesized Ni/PDCs/biomass ceramic composites exhibited a layered porous structure. This structure not only promoted the generation of a large number of heterogeneous interfaces but also improved the interfacial polarization, dipole polarization and multiple reflection. When the NiO content was maintained at 1 wt% and the filler ratio was 50 wt%, the *RL_min_* was −66.38 dB, and the *EAB* was 3.54 GHz [119]. Similarly, Wang et al. prepared NiO/BPC composites. At 16.4 GHz, the *RL_min_* was −33.8 dB, and the *EAB* was about 6.7 GHz (from 11.3 to 18.0 GHz). The extended interfaces in complex porous composites, such as the NiO–NiO, Ni–BPC and NiO–paraffin interfaces, promoted interfacial polarization and related relaxation, thereby enhancing the dielectric loss and microwave absorption properties [120].

### 3.2. Plant Fiber as Precursor

Under the trend of coordinated development of green low-carbon and high-performance materials, the exploring of electromagnetic functionalization from natural plant fibers is becoming a frontier hotspot. Wood, cotton and bamboo fibers provide unique advantages for the design of MAMs due to their rich cellulose networks, adjustable multi-level pore structure and natural dielectric response characteristics. Through carbonization modification, doping materials and process improvement, such renewable resources can accurately construct a heterogeneous interface with both dielectric loss and magnetic loss, breaking through the limitations of traditional absorbing materials in terms of lightweight, broadband absorption and environmental compatibility.

#### 3.2.1. Wood Fiber as Precursor

As the most abundant biomass material in nature, wood is mainly composed of cellulose, hemicellulose and lignin [121]. It has the advantages of high carbon content, easy availability and biodegradability. In addition, the natural directional pores of wood provide a wide range of choices for material design [122]. It is especially noteworthy that the carbonized wood can retain its original honeycomb porous structure, and the resulting carbon skeleton exhibits excellent dielectric properties and is the promising material for EMWs absorption [123,124].

**Figure 12:**
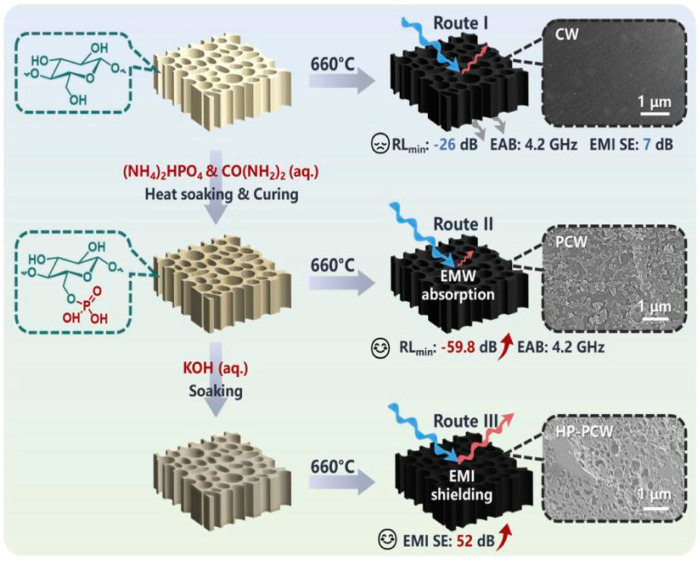
The synthesis process of CW, PCW and HP-PCW. Reprinted with permission from Ref. [125] Copyright (2024), Elsevier.

Ai et al. designed three different synthetic routes to prepare carbonized wood (CW), phosphorylated carbonized wood (PCW) and hierarchical porous phosphorylated carbonized wood (HP-PCW), respectively. As shown in Figure 12, the pure CW retained the honeycomb structure, but owing to its impedance mismatch, the *RL_min_* was only 26 dB. After being phosphorized and carbonized, the *RL_min_* significantly increased to 59.8 dB. Importantly, phosphorylation led to a large number of defects at the gas–solid interface of the PCW materials, and the interface between carbon and phosphide enhanced the interfacial polarization. In addition, the dipole polarization was enhanced under the dual influence of the dipole bond between C, P and O elements and the material defects caused by annealing. Phosphorus doping reduced the electron migration barrier of the carbon materials and enhanced the dielectric loss. At the same time, phosphoric acid and carbonized materials formed a cross-linked network to maintain pore stability and ensured the stability of the absorbing properties. Therefore, the absorbing properties were significantly optimized [125]. Zhao et al. introduced Ni particles to improve the impedance matching and magnetic loss. The wood-based porous carbon/nickel (WPC/Ni) composites were prepared by using nickel chloride and poplar as raw materials [126]. They found that when the carbonization temperature reached 700 °C, the graphitization degree increased, which increased the conductivity, dielectric loss and polarization loss of the material. At the same time, the attenuation constant increased obviously, which indicated that the electromagnetic energy conversion ability was improved. Finally, the EMWs absorption performance of WPC/Ni was the best, the *RL_min_* was −60.4 dB, and the *EAB* was 7.3 GHz. Owing to the different interface structures between Ni and graphitized carbon, polarization charges would be generated on the surface of Ni and carbon under the action of an electric field, and these charges were transferred to the surface of the material. When the EMWs reached the surface of the material, these charges moved along the surface, resulting in enhanced surface polarization.

Owing to its high porosity, unique 3D skeleton structure and ultra-low density, aerogel is considered an ideal choice for EMWs absorption materials [127,128]. Zhu et al. prepared anisotropic frameworks by the delignification of natural light wood, assembled with highly conductive few-layered Ti_3_C_2_T_x_ (f-Ti_3_C_2_T_x_) MXene by hydrogen bonding, and then obtained MXene@Wood nanocomposite aerogels by simple soaking and freeze-drying methods [129]. It was found that due to the anisotropic microstructure of the aerogels, it was easy to construct a conductive network in the parallel growth direction inside the material, thereby obtaining higher conductivity (37.04 S/m) and stronger attenuation ability. It exhibited an ideal structural carrying capacity in the vertical growth direction, which endowed the aerogel with a discontinuous conductive path and ultra-low conductivity. When the sample thickness was 3.2 mm, the *RL_min_* was −15.5 dB and the *EAB* was 8.2 GHz. Shen et al. prepared wood aerogels with natural wood as the framework and then prepared MoS_2_@Gd_2_O_3_/Mxene loaded porous carbon aerogels by self-assembly and a one-pot hydrothermal method [130]. The results show that the *RL_min_* of MGMCA was −57.5 dB and the *EAB* was 4.35 GHz at 1.9 mm ultra-thin thickness. The high-porosity characteristics of the aerogel provided a large number of interfaces, extending the propagation path of EMWs through multiple reflections and scattering, and enhancing dielectric loss and magnetic loss. However, this characteristic also led to its fragile skeleton structure, low compressive and tensile strength, and susceptibility to fracture or collapse. By compounding with biomass carbon-based materials, the mechanical strength can be effectively improved. But it may partially block the pores and reduce the absorption efficiency. Therefore, the introduction of composite materials needs to take into account the enhancement effect and pore structure retention so as to avoid the excessive sacrifice of a certain performance.

**Figure 13:**
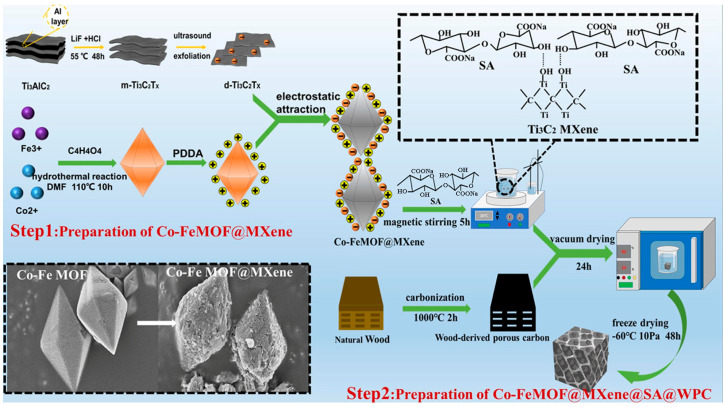
Manufacturing schematic of CoFe-MOF@Ti_3_C_2_T_x_MXene@SA@WPC. Reprinted with permission from Ref. [134] Copyright (2024), Elsevier.

**Figure 14:**
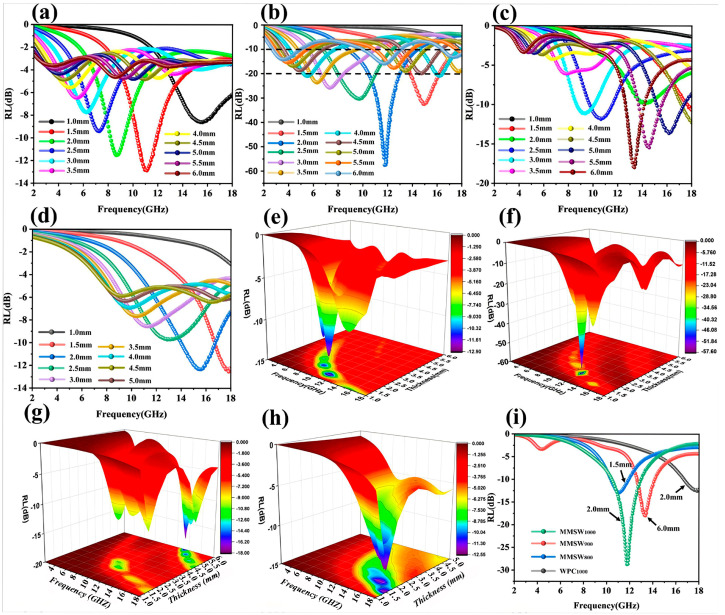
(**a**–**c**) 3D mapping of the RL values for MMSW_800_, MMSW_900_ and MMSW_1000_; (**d**) 3D mapping of the RL values for WPC_1000_; (**e**–**g**) 2D mapping of the RL values for MMSW_800_, MMSW_900_ and MMSW_1000_; (**h**) 2D mapping of the RL values for WPC_1000_; (**i**) the comparison of optimum microwave absorption properties for samples. Reprinted with permission from Ref. [134] Copyright (2024), Elsevier.

Metal–organic frameworks (MOFs) are composed of metal ions and organic ligands, which have different advantages, such as large specific surface area, adjustable microstructure and uniform composition [131]. Therefore, the construction of magnetic porous carbon composites based on MOFs has attracted extensive attention [132]. Gou et al. prepared CoNi@wood-derived porous carbon composites (WNCs) by the in situ growth of CoNi-MOF on wood and annealing at different temperatures under N_2_ atmosphere. When the annealing temperature was 700 °C and the load was 30 wt%, the *RL_min_* reached −25.96 dB at 17.98 GHz with 1.8 mm thickness [133]. Peng et al. reported on similar composites using bimetallic organic frameworks as raw materials. As shown in Figure 13, the natural wood was firstly carbonized at different temperatures (800 °C, 900 °C, 1000 °C) to obtain a wood-derived porous carbon skeleton (WPC) as well as fully mixed CoFe-MOF and Ti_3_C_2_T_x_MXene. After adding biocompatible sodium alginate (SA) solution, the CoFe-MOF@Ti_3_C_2_T_x_MXene precursor was prepared by self-assembly. Finally, the CoFe-MOF@Ti_3_C_2_T_x_MXene@SA@WPC (MMSW) foam was obtained by using highly ordered honeycomb cells in the WPC skeleton as microreactors [134]. Under 900 °C carbonization, the composites with −57 dB of *RL_min_* and 5.8 GHz of *EAB* were obtained at 1.5 mm thickness (Figure 14). In both studies, CoNi and CoFe metal frames were typical magnetic materials, which can cause magnetic loss (eddy current loss and magnetic resonance). Secondly, the carbonized 3D carbon skeleton can be used as a conductive network for electron hopping and migration, resulting in strong conduction loss. In addition, rich interfaces, carbon defects and heteroatom-induced dipole polarization contributed to enhancing the dielectric loss. However, the difference is that the latter mixed Ti_3_C_2_T_x_MXene nanosheets with MOF, and they used their surface electronegativity to make the MOF surface rough, bringing more non-uniform interfaces and enhancing interface polarization. In addition, they provided abundant charge carriers, resulting in stronger dielectric loss. It is worth noting that CoFe-MOF@Ti_3_C_2_T_x_MXene and WPC were grafted on the porous walls of the WPCs, which strengthened the connection between adjacent cells, enriched the conductive structure of the 3D network, and helped to attenuate the EMWs energy. Therefore, MMSW exhibited better microwave absorption performance when the material thickness was thinner.

**Figure 15:**
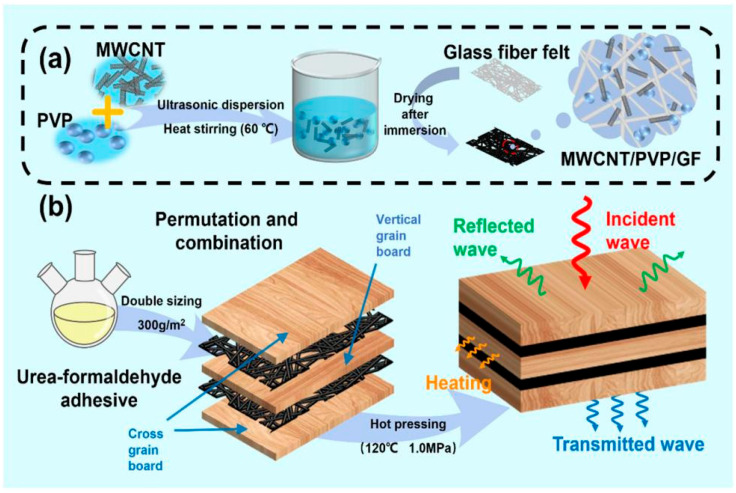
(**a**) The preparation process of MPG depletion layer; (**b**) the Flow chart of synthesis of MPG@RAS composites. Reprinted with permission from Ref. [136] Copyright (2024), Elsevier.

It is a common and effective preparation method to introduce CNT with a cavity structure into solid carbon materials. Xiao et al. reported Fir@Co@CNT biomass carbon composites using Fir, Co(NO_3_)_2_ and melamine as raw materials. Through high-temperature carbonization, the 1D CNTs with a large aspect ratio and high anisotropy were load on the surface of 3D wood carbon pores [135]. The *RL_min_* of Fir@Co@CNT was −52 dB at 10.72 GHz, and the *EAB* was 4.2 GHz. The *EAB* covered the entire C-band, X-band and Ku-band. Peng et al. introduced multi-walled carbon nanotubes (MWCNTs) to prepare MWCNT/PVP/GFM@RAS (MPG@RAS) composites, and the corresponding schematic is shown in Figure 15 [136]. Here, the structure completely covered the entire Ku-band, and the *RL_min_* was −40 dB at 13.4 GHz. From the perspective of the absorption mechanism, first of all, with the increase in CNT content, it was helpful to construct a rich conductive network. Secondly, the defects and functional groups in CNTs caused the charge to deviate from the charge center and generate dipoles, forming polarization sites. These all contributed to the enhancement of the microwave-absorbing properties. Notably, compared with the latter, the former introduced magnetic Co particles, which loaded on the surface of CNTs induced interfacial polarization and further enhanced the EMWs loss ability. However, the latter paid more attention to the multifunctional ability of microwave-absorbing materials and proposed a lightweight and broadband design to solve the common problems of excessive thickness and high density, which were usually related to traditional device configuration. In addition, according to the latest research findings, Zhang et al. developed a transparent wood composite with ultra-strong EMWs absorption and optical properties. The work firstly used NaClO_2_ for delignification and then infiltrated pre-polymerized polyacrylamide (PAM) containing a small amount of CNT, silver nanowires and reduced graphene oxide (RGO) to prepare transparent wood composites [137]. At the same time, the authors used epoxy resin, polystyrene (PS), polymethyl methacrylate (PMMA) or polydimethylsiloxane (PDMS) instead of PAM to prepare a series of transparent wood. By comparison, the CW-PAM/filler wood composite had better EMWs absorption performance and higher light transmittance when the thickness was 2.0 mm. In the frequency range of 8.2~18 GHz (X-band and Ku-band), the best *EAB* reached 9.5 GHz. In addition to the above reports, Dong et al. prepared a natural wood-derived 3D carbon skeleton by a simple KOH activation and carbonization and then introduced NiCo_2_S_4_ nanosheets into the 3D carbon skeleton through a simple surfactant-assisted hydrothermal process to successfully prepare NiCo_2_S_4_/C hybrids [83]. When the sample thickness was 1.91 mm, the *RL_min_* reached −64.74 dB. The *EAB* was 5.26 GHz, ranging from 9.22 to 14.48 GHz. When the thickness was 2.23–2.31 mm, the entire X-band was covered. Due to the high conductivity of NiCo_2_S_4_, the conductivity of the 3D conductive network derived from natural wood was enhanced, which improved the dielectric absorption capacity.

Although natural wood fiber-derived carbon-based MA materials have received significant attention and fast development, they still face some challenges regarding their EMWs absorption performance. Maintaining the mechanical strength after carbonization at a specific level is difficult, which requires innovative solutions in sample pretreatment, heating procedures, and environmental conditions. The harsh carbonization steps limit the preparation of highly conductive materials. The carbon content of cellulose-based carbon materials is generally low, while the lignin structure is complex, the degree of graphitization is high, and the treatment is difficult, which affects its conductivity and corresponding conductivity loss capacity. In addition, wood-based carbon materials have poor flexibility in the processing process, which hinders the further optimization of their porous structure. Therefore, one of the future research directions is to adjust the intrinsic properties of wood fiber-derived carbon by improving the processing technology.

#### 3.2.2. Cotton Fiber as Precursor

As a common plant biomass material, cotton is mainly composed of cellulose, which is naturally rich in N and O elements [138]. The cotton-derived carbon fibers have a unique one-dimensional structure and anisotropy as well as high aspect ratio. This not only endows the material with excellent scalability and flexibility but also helps to form carrier transport paths and enhance dielectric loss [139]. However, the pure cotton-derived carbon fibers have poor impedance matching and a single loss mechanism, which cannot meet the needs of practical applications. Thus, the exploring of cotton-derived carbon fiber-based composites is vital [140].

**Figure 16:**
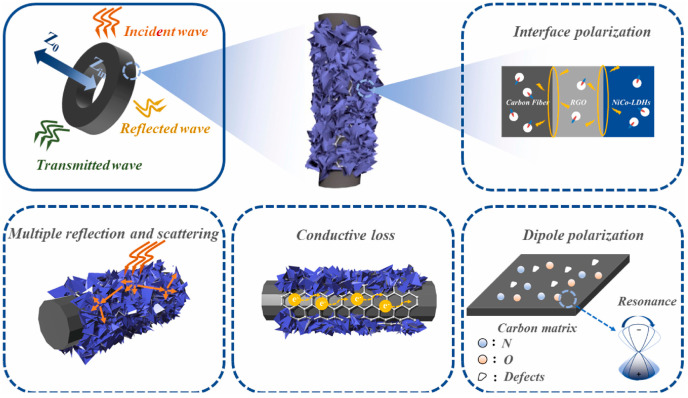
The microwave absorption principle diagram of CF/RGO/LDH composites. Reprinted with permission from Ref. [144] Copyright (2022), Elsevier.

Recently, the composites based on cotton-derived carbon fiber with novel microstructure and multi-component have received significant attention. Li et al. reported on the microwave-assisted synthesis of Fe@nanoporous carbon@carbon fiber (Fe@NPC@CF) composites [141]. When the matching thickness was 2.5 mm, the filling rate was as low as 25 wt%, the *RL_min_* was −46.2 dB, and the *EAB* was 5.2 GHz. Some researchers introduced CNTs to change the microstructure of the absorbing materials. Yang et al. used cotton and a zeolitic imidazolate framework-67 to prepare composites loaded with Co particles and wrapped with CNTs [142]. The product displayed ultra-low apparent density (0.0198 g/cm^3^). At 7.8 GHz and 2 mm sample thickness, the *RL_min_* and *EAB* reached −53.5 dB and 8.02 GHz, respectively. Fan et al. synthesized CF@Ni@CNT MA materials by a simple freeze-drying method using Ni particles instead of Co particles [143]. The helical CF@Ni@CNT fiber exhibits high thermal conductivity (4.27 W/(m·K)), high absorption bandwidth (7.52 GHz) and lower load (10 wt%), showing excellent comprehensive performance. Lu et al. successfully synthesized a layered core–sheath composite (CF/RGO/LDH) by integrating CFs, reduced RGO and NiCo-layered double hydroxide (NiCo-LDH) through electrostatic self-assembly and solvothermal methods [144]. The ternary CF/RGO/LDH composites exhibited good MA properties. When the filling rate was 20 wt% and the thickness was 2.5 mm, the *RL_min_* was −60.9 dB at 10.3 GHz, and the *EAB* was 6.1 GHz; the microwave absorption mechanism is shown in Figure 16. Firstly, cotton was carbonized at high temperature, which provided a large number of heterogeneous atomic defects for polarization effect. They acted as polarization factors to promote multiple relaxations, thereby enhancing the dielectric loss ability of the CF. Secondly, RGO acted as an intermediate layer to accelerate the directional migration of internal electrons and enhanced the conduction loss of the composites. At the same time, 2D NiCo-LDH sheets were regularly stacked on the surface of the CF/RGO, forming abundant small voids. The incident EMWs were scattered and reflected several times inside the material, which increased the EMWs loss. In addition, the CF/RGO/LDH composites had many heterogeneous interfaces, resulting in the generation of interfacial polarization, which should not be ignored regarding its ability to to improve the microwave loss capacity. Similarly, Zhang et al. prepared heterogeneous RGO/Ni/C composites; a corresponding *RL_min_* of −39.3 dB and *EAB* of 4.6 GHz were obtained [145].

**Figure 17:**
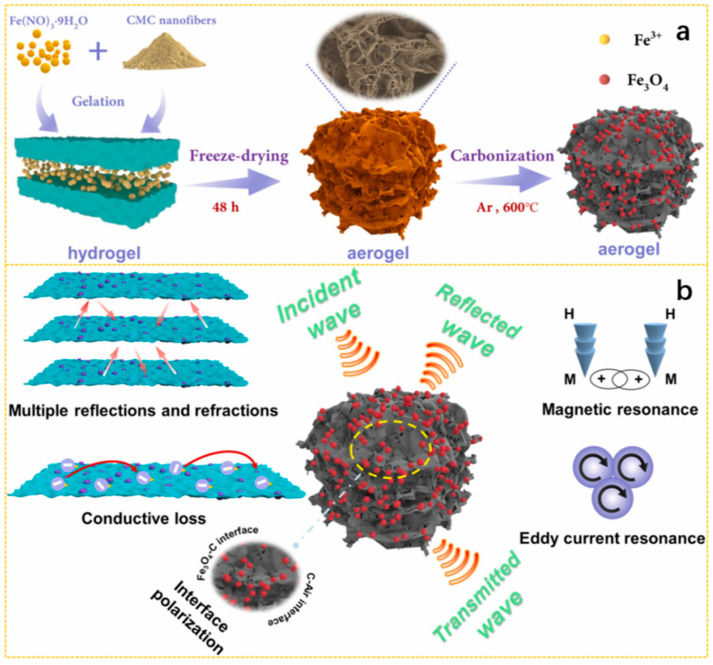
(**a**) Preparation process of Fe_3_O_4_@PC composites; (**b**) microwave absorption principle diagram of Fe_3_O_4_@PC material. Reprinted with permission from Ref. [146] Copyright (2024), Elsevier.

Yang et al. designed Fe_3_O_4_@porous carbon (Fe_3_O_4_@PC) composites using CF as a carbon source by integrating freeze-drying and carbonization processes; the composition diagram is shown in Figure 17a [146]. When the match thickness was 2.9 mm, the *RL_min_* was −54.69 dB at 12.72 GHz and the *EAB* was 7.72 GHz. The microwave-absorbing mechanism of Fe_3_O_4_@PC in Figure 17b shows that the porous structure enhanced the multiple reflection and refraction of EMWs. At the same time, the magnetic Fe_3_O_4_ nanoparticles acted as a polarization center, causing polarization relaxation and interfacial polarization under the action of the applied electric field. In addition, due to the difference in conductivity between carbon and Fe_3_O_4_, the charge accumulated at the heterogeneous interface, causing polarization loss and significantly increasing the dielectric loss. Fe_3_O_4_ improved the magnetic loss of the absorbing materials. The synergistic effect of these mechanisms led to the excellent microwave-absorbing performance of the Fe_3_O_4_@PC composites. Yin et al. successfully synthesized CF@H-Fe_3_O_4_/CoFe composites combining simple hydrothermal and calcination [147]. The sample exhibited an *RL_min_* of −40.15 dB at 0.71 GHz and −40.85 dB at 0.59 GHz with thicknesses of 3 mm and 3.5 mm, respectively. In the work, the magnetic nano Fe_3_O_4_ particles acted as polarization centers to induce polarization relaxation and interfacial polarization loss. In addition, the charge accumulation at the heterogeneous interface led to polarization loss, which significantly increases the dielectric loss due to the difference in conductivity between CF and Fe_3_O_4_. Li et al. prepared novel NiFe_2_O_4_/Carbonized cotton fiber (NiFe_2_O_4_/CCF) by doping different proportions of NiFe_2_O_4_ particles with CF as the carbon source and then adjusting the electromagnetic parameters. When the load was 42.3 wt%, the *EAB* reached 6.5 GHz, and the *RL_min_* was up to −45.3 dB [148].

MoS_2_ is also one of the potential candidates for microwave-absorbing materials due to its 2D structure, high specific surface area and ultra-thin thickness. Chen et al. used MoS_2_ to modify cotton fiber derived carbon (CFC) to prepare CFC/MoS_2_ composites with a hollow tube carbon fiber structure [140]. When the atomic ratio of molybdenum to carbon (Mo:C) in the current-driven solution was 3:20, the EMWs absorption effect of CFC/MoS_2_ was the best. At 9.52 GHz and 3.0 mm, the *RL_min_* reached −49.7 dB and the *EAB* reached 3.6 GHz (8.0–11.6 GHz). By adjusting the proportion of MoS_2_, the impedance matching was effectively improved.

Aerogel is receiving increasing attention due to its special porous and light character; some researchers have reported its ability to be used as a microwave absorption material. He et al. successfully prepared lightweight TiO_2_@C/carbon fiber aerogels using cotton as a carbon source and combining with Ti_3_C_2_T_x_ [149]. The ideal performance was obtained; the *RL_min_* reached −43.18 dB and the *EAB* reached 4.36 GHz. In the aerogel, the 3D interconnected porous network created an abundant interface. The unbalanced charge distribution accumulated around the interface, resulting in a large number of dipoles gathered. Under the action of the applied electromagnetic field, the charge migrated through the interface, and the interface polarization was enhanced.

Based on the above discussion, the natural cotton fiber-derived carbon microwave absorption materials have a bright future. The carbonized cotton fiber has a hollow porous structure, which provides more opportunities for multiple scattering and the reflection of EMWs. Its high specific surface area provides a large number of attachment points for the load material and enhanced the interface polarization effect. However, cotton fiber-derived carbon composites still have some shortcomings. In particular, the contradiction between the hollow structure, high porosity and mechanical properties is an urgent problem that needs to be solved. In the future, we can solve this contradiction by the following strategies:

① Magnetic nanomaterial composite: Fe_3_O_4_, Co, Ni and other particles were introduced to induce interfacial polarization and multiple scattering to enhance the absorbing properties. At the same time, the fiber gap was filled to inhibit crack propagation and improve the mechanical strength.

② Carbon nanomaterial composite: carbon nanotubes and graphene were used to construct a heterogeneous interface, optimize the dielectric loss and fracture toughness, and form a surface strengthening layer.

③ Hierarchical pore control: Multi-stage KOH activation constructs a ‘micropore (induced dipole polarization)–mesoporous (enhanced mechanical properties)–macropore (increased microwave propagation distance)’ gradient structure, synergistically enhancing compressive strength and electromagnetic loss efficiency.

#### 3.2.3. Bamboo Fiber as Precursor

Bamboo is a very rich biomass resource in Asia and South America. It grows very fast and can be used sustainably after one afforestation [150]. It has been widely used in industry, agriculture and biology fields, and so on. Over the past few years, the application of bamboo in the field of microwave absorption has attracted more and more attention. The porous structure of bamboo parenchyma is beneficial to improve the dielectric constant, reflection, scattering ability and ability to carry other particles of the composites [151].

Han et al. extracted cellulose from waste bamboo and prepared a series of biochar-based porous sheets with different pore sizes by pyrolysis and chemical etching [152]. When the etching time was 6 h, the material had good electromagnetic properties. Its *RL_min_* reached −15.8 dB at 1.6 mm and 17.4 GHz, and the *EAB* reached 3.8 GHz at 1.7 mm. Pang et al. and Li et al. used a similar process to prepare bamboo-derived BC/FeCo and CoFe alloy/carbonized bamboo fiber composites, respectively. The former achieved a strong reflection loss of more than −40 dB at a high frequency of 14.1 GHz (matching thickness of 1.9 mm) and a low frequency of 5.5 GHz. At 3.0 mm, 4.7 GHz of *EAB* was achieved, ranging from 6.6 GHz to 11.3 GHz. When the nitrate concentration was 0.2 mol/L and the calcination temperature was 700 °C, the sample prepared by the latter had good electromagnetic properties. The *RL_min_* was up to −42.74 dB with 2.7 mm thickness, and the *EAB* reached 5.7 GHz. The enhanced microwave-absorbing properties were inseparable from the introduction of CoFe particles. Loading CoFe magnetic particles on carbon fibers provided rich interfaces and defects, which greatly promoted interfacial polarization and dipole polarization. In addition, the introduction of CoFe particles endowed the composites with a magnetic loss mechanism, which also consumed EMWs energy through the natural resonance and exchange resonance [151,153].

**Figure 18:**
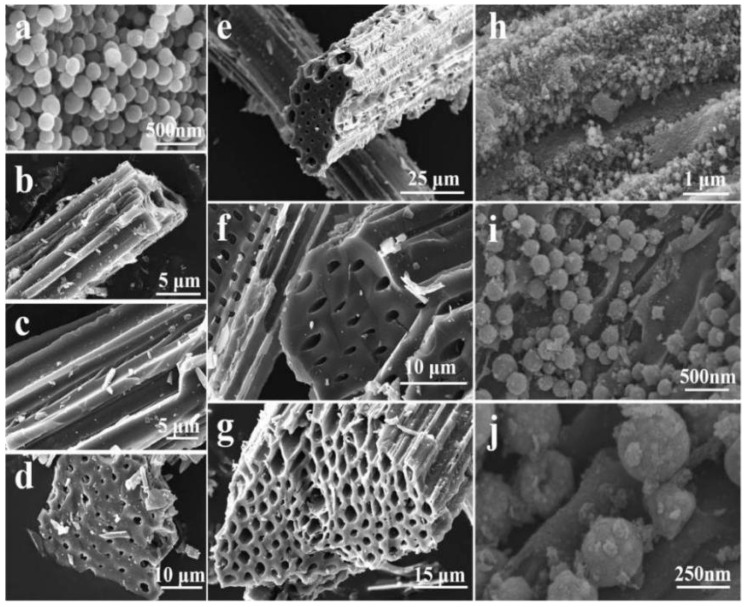
SEM images of (**a**) CoNi-MOF precursor; (**b**,**c**) BF; (**d**) A-CBF; (**e**–**g**) ABF; (**h**–**j**) CN-ABF. Reprinted with permission from Ref. [154] Copyright (2021), Elsevier.

Zhao et al. prepared carbonized pure bamboo fiber (BF), activated bamboo fiber (ABF), activated/carbonized bamboo fiber (A-CBF), and CN-ABF composites compounding ABF with CoNi-MOF alloy, respectively [154]. From Figure 18e–g, it can be seen that the ABF exhibits rich and uniform pores, which greatly increases the surface area of carbon fiber. However, A-CBF (Figure 18d) prepared by activation first and then carbonization displayed very narrow, small and uneven pores, indicating the preparation technology had an important effect on the microstructure of carbon fiber. The CN-ABF composites were prepared by a simple hydrothermal method. The results showed that CoNi-MOF particles were uniformly loaded on bamboo-derived carbon fibers (Figure 18h–j). The CN-ABF composites exhibited excellent microwave absorption performance. When the sample thickness was 2.66 mm, the *RL_min_* reached −75.19 dB at 11.12 GHz, and the corresponding *EAB* was 4.56 GHz. The reason why CN-ABF had better absorption performance was that the increase in porosity enhanced the charge polarization and multi-layer interface polarization. In particular, the introduction of CoFe magnetic particles further improved the magnetic loss and optimized the impedance matching of CN-ABF composites.

**Figure 19:**
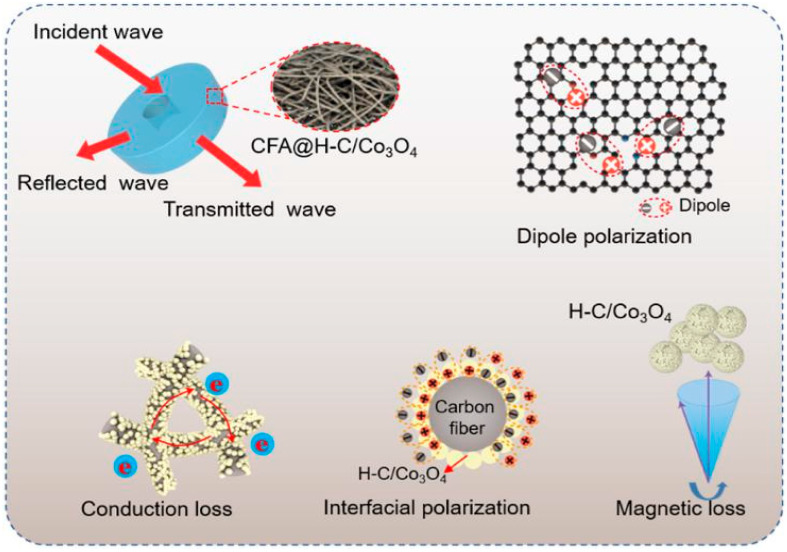
The schematic diagram of the microwave absorption mechanism of CFA@H-C/Co_3_O_4_. Reprinted with permission from Ref. [157] Copyright (2024), Elsevier.

In addition to magnetic materials, the metal oxides also can affect the microwave-absorbing properties of carbon-based composites [155]. Zhang et al. synthesized the biocarbon/ferrite material with ferrite particles into the inner cavity of the bio-carbon through low-temperature carbonization and impregnation; the composite showed an *RL_min_* of −43.2 dB and an *EAB* of 4.72 GHz with 2.0 mm matching thickness [156]. Chen et al. constructed lightweight carbon fiber aerogel@hollow-carbon/cobalt oxide (CFA@H-C/Co_3_O_4_) composites by in situ chemical deposition and pyrolysis using ZIF-67, Co_3_O_4_ and bamboo cellulose-derived carbon fibers as raw materials. The microwave absorption mechanism of CFA@H-C/Co_3_O_4_ is shown in Figure 19. Co_3_O_4_ showed high saturation magnetization, which can effectively improve the Snoek limit, thereby improving the EMWs absorption performance in the X-band and Ku-band [157,158]. The introduction of high-resistivity hollow carbon/Co_3_O_4_ in the carbon fiber network effectively reduced the skin effect, promoted EMWs to enter the composites, and ensured good impedance matching. At the same time, a large number of effective interfaces were formed between carbon fiber and hollow carbon/Co_3_O_4_, which was beneficial to the interfacial polarization. Under a low filling rate (15 wt%), when the thickness was 3.0 mm, the *RL_min_* reached −43.5 dB at 12.88 GHz, and the *EAB* reached 7.84 GHz, covering most of the X and Ku bands.

**Figure 20:**
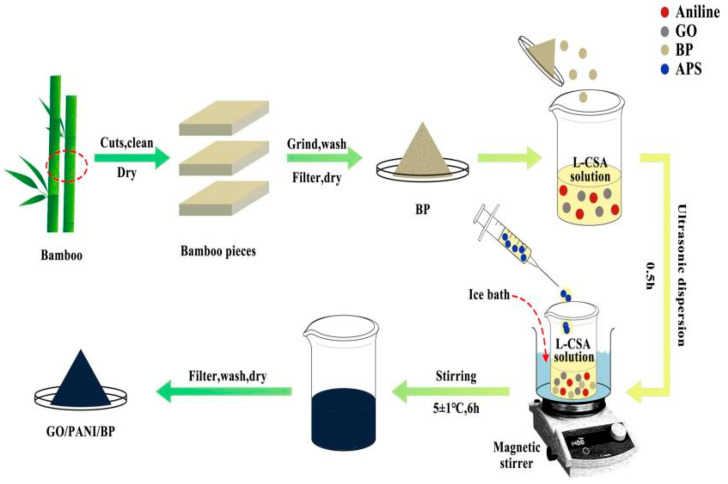
The preparation process of GO/PANI/BP composites. Reprinted with permission from Ref. [161] Copyright (2024), Elsevier.

PANI as a conductive polymer material was also used to prepare microwave-absorbing materials due to its design flexibility, reduced microwave loss and corrosion resistance [159]. However, the high conductivity and serious impedance mismatch of pure PANI requires sacrificing a certain amount of lightweight to achieve improved microwave absorption performance. To address this issue, Wu et al. prepared GO/PANI/BP composites based on the scheme in Figure 20. In the work, graphene oxide (GO) was used as the growth template of PANI due to its rich oxygen-containing functional groups, special hydrophilicity, dispersion and wave-transparent properties [160]. And then the GO/PANI composites were loaded on the surface of natural bamboo powder (BP) by interfacial polymerization technology [161]. BP brought more electromagnetic scattering and reflection channels, and it enhanced the dielectric storage and depletion capabilities through a combination of GO and PANI. Additionally, the coupling between N in PANI and the carbon skeleton of GO increased the dipole polarization. The *RL_min_* of GO/PANI/BP was −44 dB at 9.36 GHz and 3 mm thickness, and the *EAB* was 5.36 GHz at 2 mm thickness.

Silicon carbide (SiC) has become a promising EMWs absorbing material due to its excellent physical and chemical properties (such as high specific surface area, corrosion resistance, good thermal stability and adjustable conductivity). Zheng et al. used bamboo charcoal and silica as raw materials to prepare beaded SiC/SiO_2_ nanowires by the carbothermal reduction method [162]. The complex 3D network, large specific surface area and larger interface had a great influence on the microwave absorption performance. The product’s *RL_min_* and *EAB* reached −43.58 dB and 2.32 GHz, respectively.

Although bamboo fiber-derived carbon composites have achieved excellent results in the field of EMWs absorption, there are still challenges in terms of cost and performance. First of all, although the acquisition cost of bamboo itself was very low, the preparation cost of composites increased due to the high preparation cost of carbon fiber. Secondly, most bamboo fiber-derived carbon-based materials exhibit good performance in the middle- and high-frequency bands due to their excellent dielectric loss properties, while the performance at low frequencies still needs to be improved. In addition, the function of absorbing materials is single, and most materials only focus on optimizing absorbing properties, ignoring the application in actual environment (whether it has a certain ability to resist external forces). Therefore, more in-depth research is needed in order to develop microwave-absorbing materials with low cost, low frequency absorption capacity and various functions.

### 3.3. Other Biomass Materials as Precursors

In recent years, other biomass-based composites with excellent properties have also received extensive attention: for example, algae, fruits, straw, etc. These materials are easy to be compounded with other materials, which has great potential in the field of absorbing.

**Figure 21:**
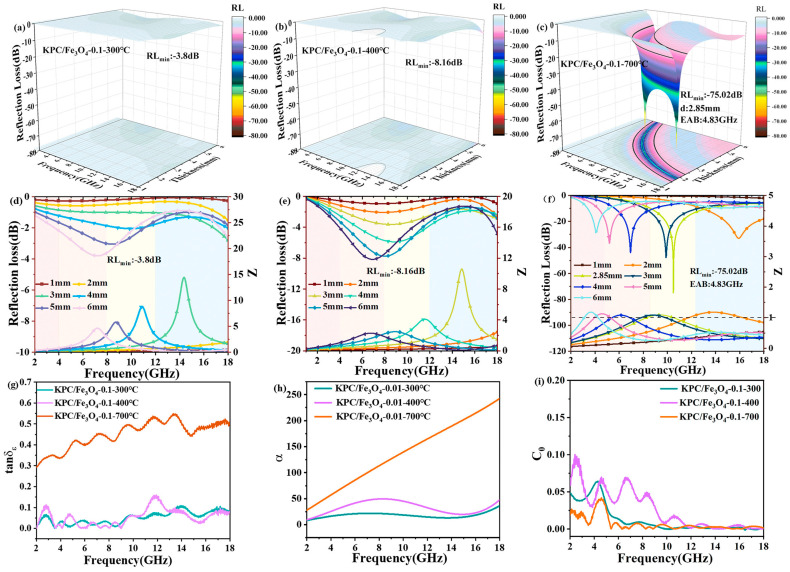
Reflection loss curves of composite at different thicknesses (**a**) KPC/Fe_3_O_4_-0.1–300 °C; (**b**) KPC/Fe_3_O_4_-0.1–400 °C; (**c**) KPC/Fe_3_O_4_-0.1–700 °C composite; reflection loss, impedance matching of the (**d**) KPC/Fe_3_O_4_-0.1–300 °C; (**e**) KPC/Fe_3_O_4_-0.1–400 °C; (**f**) KPC/Fe_3_O_4_-0.1–700 °C; (**g**) dielectric loss tangent tanδ_ε_; (**h**) attenuation constant α; (**i**) C_0_ valuesof KPC/Fe_3_O_4_-0.1. Reprinted with permission from Ref. [164] Copyright (2024), Elsevier.

Wang et al. prepared porous Ni@BPC composites by the solvothermal method using laver as a carbon source [163]. When the mass ratio of Ni to BPC was 1:3, it showed an *RL_min_* of −35.73 dB at 3.0 mm and *EAB* of 6.37 GHz (from 10.35 to 16.72 GHz) at 2.5 mm. This was attributed to the unique 3D porous structure of Ni@BPC and the large number of interfaces between BPC and Ni. Liu et al. proposed using kelp as a carbon source and FeCl_3_·6H_2_O as a raw material to prepare kelp-derived 3D porous carbon/Fe_3_O_4_ (KPC/Fe_3_O_4_) composites. From Figure 21, under 700 °C carbonization, the KPC/Fe_3_O_4_-0.1 sample possessed the best microwave absorption properties, which were −75.02 dB for *RL_min_* and 4.83 GHz for *EAB*, respectively. The outstanding properties were attributed to two aspects: one was that the addition of magnetic Fe_3_O_4_ particles provided a double depletion mechanism. The other was that the rich interface of porous carbon materials formed a large number of attachment points, which were beneficial to enhance the interface polarization and relaxation loss [164]. Yu et al. prepared rose-derived carbon/Co (RC/Co) and rose-derived carbon/Ni (RC/Ni) MAMs by simple impregnation and one-step carbonization using rose-derived carbon as raw materials and combining Co and Ni nanoparticles. At a matching thickness of 1.58 mm, the *RL_min_* of RC/Co was −47.89 dB at 13.60 GHz, and the *EAB* was 4.08 GHz, while the *RL_min_* of RC/Ni was −45.36 dB at 12.88 GHz, and the *EAB* was 3.02 GHz at 1.56 mm thickness. The array structure of rose petals remained intact after carbonization, and its internal wrinkles and porous structure reduced the density and increase the interface. At the same time, the introduction of Co and Ni magnetic nanoparticles led to enhanced magnetic loss, synergistically optimized the dielectric-magnetic loss capacity, and improved the EMWs attenuation performance [165].

**Figure 22:**
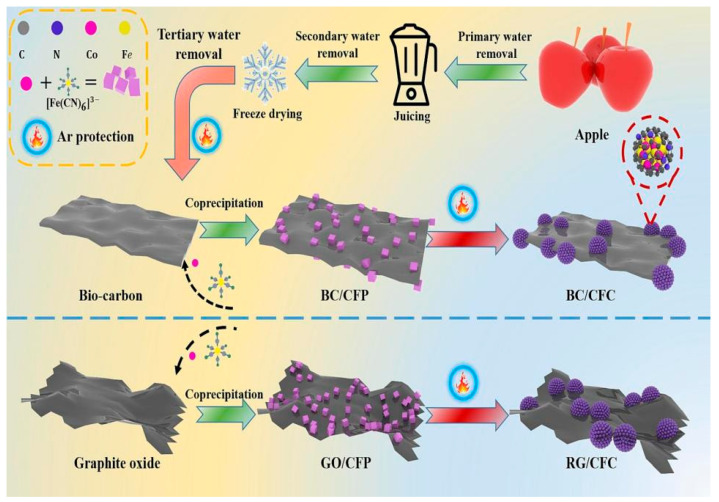
Illustration of the synthetic process of BC/CFC and RG/CFC. Reprinted with permission from Ref. [168] Copyright (2023), Elsevier.

Huang et al. anchored CeO_2_ on the surface of porous carbon (PC) by simple hydrothermal and pyrolysis methods using pine cones as the carbon source. The characterization results showed that when the cerium salt content was 0.6 mmol, the *RL_min_* reached −56.04 dB and the *EAB* was 5.28 GHz. During the synthesis process, when Ce^4+^ was converted to Ce^3+^, many oxygen vacancies were generated, which were beneficial to electron migration, increasing the generation of charge relaxation, and then improving the EMWs attenuation [166]. Wen et al. developed a green one-step carbonization route to convert waste coffee grounds into a porous C/Fe mixture with a high carbonization rate. The *RL_min_* was −52.68 dB, and the *EAB* was 6.40 GHz at a thickness of 3.0 mm. The MA performance was better than that of most biomass carbon, and the enhancement was due to the synergy of the aporous carbon and Fe nanoparticles [167]. Wang et al. synthesized biocarbon/CoFe-PBA (BC/CFP) and GO/CoFe-PBA (GO/CFP) by a co-precipitation method with Prussian blue analogues (PBA) based on apple-based derived carbon materials and reduced graphene oxide (rGO), respectively. After that, BC/CFP and GO/CFP were annealed at different temperatures for 2 h to prepare biocarbon/CoFe@C (BC/CFC) and rGO/CoFe@C (RG/CFC). The composite schematic is shown in Figure 22. Under the same conditions, the absorbing performance of the former is better than that of the latter. When the matching thickness was 1.62 mm, the *RL_min_* was −72.57 dB, which meant that 99.99999% of the EMWs were attenuated. An *EAB* of 5.25 GHz was obtained at 1.61 mm. The reason was that the biocarbon-based composites have better impedance matching, which made it easier for more EMWs to enter the interior and be consumed. At the same time, it contained a large amount of pyridine-n, which provided more dipole polarization [168].

In many areas, straw is regarded as agricultural waste, which is usually used as fuel, fertilizer or feed [169]. Obviously, it has not been fully applied as a natural biomass resource with low cost, wide source and high yield. Because of its inherent porous structure, it is receiving attention from material researchers. They are tapping the potential application of straw and turning waste into treasure. Yin et al. successfully loaded Fe and Ni dual magnetic particles onto sorghum straw-derived carbon by a two-stage calcination process. The prepared composites had excellent low-frequency absorption properties due to the synergistic effect between multi-reflection and scattering caused by the porous structure, polarization loss caused by multiple interfaces on the surface of carbon materials and magnetic loss caused by magnetic particles [170]. At 600 °C, the *RL_min_* was −44.18 dB at 0.49 GHz. At 700 °C, the *RL_min_* at 0.81 GHz reached −46.36 dB. Using wheat straw as the carbon source, Li et al. successfully anchored Co particles on the surface of carbon black (CB) by a simple thermal reduction method. The CB/Co@C composite was successfully prepared [171]. The magnetic properties and EMWs absorption properties were adjusted by changing the filling amount of Co particles. When the concentration of Co^2+^ was 0.02 mol/L, the CB/Co@C obtained the best *RL_min_* (−53.99 dB). When the concentration was 0.05 mol/L, the widest *EAB* (6 GHz, 6.72–12.72 GHz) was reached. Zhou et al. used a similar method to prepare a porous biocarbon/NiCo absorber by combining magnetic NiCo alloy with straw-derived carbon [172]. The magnetic NiCo alloy enhanced the dielectric loss through interfacial polarization At a thickness of 2.2 mm, the *RL_min_* was −27.0 dB and the *EAB* was 4.4 GHz. Chen et al. successfully prepared composite absorbing materials by a soaking-pyrolysis method using waste corn straw core as the precursor and FeCl_3_ as the iron source. At a thickness of 3.5 mm, the *RL_min_* reached −30.03 dB, and the corresponding *EAB* was 4.17 GHz [173].

For the convenience of the readers’ comparison, this review listed the EMWs absorption materials and their performance parameters from the representative references reported, which are listed in Table 1.

## 4. Summary and Outlook

Biomass-based microwave absorption materials have attracted more and more attention in the field of electromagnetic absorption due to their unique characteristics such as lightweight, environmental friendly, economy and renewability. This article summarized and analyzed the progress of biomass-derived carbon-based microwave absorption materials in detail. It discussed the application of different types of biomass-derived carbon-based materials. By selecting different kinds of biomass raw materials, microwave absorption absorbers with different microstructures were designed to improve and enhance the impedance matching and attenuation ability. Herein, plant shells, plant fibers and other potential biomass materials-derived carbon and their composites were discussed. Compared with traditional microwave absorption materials, the remarkable properties of biomass-derived carbon-based absorbers has been confirmed, such as anisotropy, high porosity, high specific surface area, rapid preparation, high heating efficiency and good composite effects, which endowed the absorbers with flexibility, lightweight and versatility. Biomass-derived microwave absorption materials provided new ideas for researchers to prepare new multifunctional composites. The microwave absorption properties were adjusted by changing the composition, morphology and design process of biomass-based composites.

At present, the best biomass structure was ‘microporous–mesoporous–macroporous’, which can synergistically improve the comprehensive performance of materials by inducing polarization loss, enhancing mechanical strength and prolonging the EMWs path, respectively. By compounding with magnetic particles, the composites exhibited more significant absorbing properties. The biomass carbon skeleton provides polarization relaxation loss through dielectric loss, and the magnetic component generates magnetic loss through natural resonance. In addition, after summarizing and analyzing various biomass materials, it was found that the biomass material with the best available performance was the lignocellulose composite absorbing material. The material exhibited high *RL_min_* numerical characteristics, indicating that it has a strong ability to absorb energy at a specific frequency point, and its wide *EAB* response range verified the broadband adaptability of the absorber in multi-band applications. At the same time, with abundant resource sources and low processing cost, wood showed broad research prospects and industrialization potential.

Different biomass-derived carbon sources exhibited various structure and morphology, which provided the rich design strategies for high-performance microwave absorption materials. The above discussions have indicated that biomass-derived carbon-based microwave absorption materials have bright development prospects and application space. However, it is also worth considering that there are few reported materials that can fully meet all performance requirements currently. Thus, more selections and explorations should be carried out:

(1) Comprehensiveness: In the current research field, the research objects of biomass absorbing materials mainly focus on natural materials grown on land, such as wood, cotton, bamboo and so on. Although these materials have good absorbing properties, they have problems such as long growth cycle and relatively poor structural properties. It is worth noting that the marine area is rich in biomass resources, such as seaweed, shells, sponges, etc. These materials have significant advantages such as fast growth rate, superior structural performance, and easy access. In the future, researchers can conduct in-depth research on these characteristics of marine biomass materials.

(2) Material stability: According to the current research, the surface structure of biomass-derived carbon materials is easily affected by the external environment, chemical treatment and other factors, resulting in irreversible changes in the structure. How to ensure the stability of the surface structure during long-term use through a reasonable synthesis process and post-treatment technology is a key problem to be solved.

(3) Material uniformity: When loading materials on biomass-derived carbon, the existing loading methods may lead to an uneven distribution of loading materials due to factors such as the surface characteristics of the carrier, concentration of solution, and temperature. How to realize the uniform distribution of the load material and avoid agglomeration is one of the future research directions.

(4) Environmental adaptability: Although biomass-based absorbing materials have attracted much attention in the research field of microwave absorption materials due to their renewability, environmental friendliness and potential excellent properties, most biomass-based absorbing materials still show the limitations of adaptability in the face of complex and changeable environmental conditions. This limitation is mainly reflected in the fact that it is difficult to maintain the structural stability and microwave absorption performance in harsh conditions such as high pressure, high temperature and strong corrosion in specific or extreme environments. These external stresses will limit the performance of absorbing materials and have a huge impact on their service life. In the future, how to ensure the high stability of biomass-based absorbing materials in harsh environments is not only an urgent technical problem to be solved but also the key to promoting the sustainable development of this field.

## Figures and Tables

**Table 1:** The comparison of properties of various biomass-based EMWs absorption materials.

Biomass Material Category	Biomass Feedstock	Material Components	*RL_min_* (dB)	*EAB* (GHz)	Ref.
Plant shells	Rice husk	Ni/porous carbon(PC)	−58.50	3.51	[88]
		NiCo_2_/C	−55.62	3.60	[89]
		FeCo/C	−68.11	3.76	[90]
		ZnO/NiCo/C	−52.50	4.48	[92]
		Fe_3_O_4_/C	−52.14	13.00	[94]
	Coconut shell	TiP_2_O_7_/C	−32.40	6.00	[98]
		Coconut shell/epoxy resin	−23.50	-	[99]
		Fe/Fe_3_C	−48.87	7.94	[107]
		Biomass-derived Borocarbonitride/natural rubber	−54.24	4.16	[108]
	Almond wood shell	C/Fe_x_O_y_	−37.90	7.04	[112]
	Walnut shell	PNT-Carbonized walnut shell-FeCl_3_ Activated material	−67.60	5.40	[116]
		Fe_3_O_4_@C/C	−56.61	5.68	[117]
	Peanut shell	PANI/biomass porous carbon(BPC)	−40.89	4.24	[114]
		Ni/polymer-derived ceramic/biomass ceramic	−66.38	3.54	[119]
	Pine nut shell	C@NiCo-LDHs@Ni aerogel	−57.40	6.40	[118]
Plant fiber	Wood fiber	Wood-based porous carbon (WPC)/Ni	−60.40	7.30	[126]
		MoS_2_@Gd_2_O_3_/Mxene	−57.50	4.35	[130]
		CoFe-MOF@Ti_3_C_2_T_x_MXene@SA@WPC	−57.00	5.80	[134]
		Fir@Co@CNT	−52.00	4.20	[135]
		NiCo_2_S_4_/C	−64.74	5.26	[83]
	Cotton fiber	Fe@nanoporous carbon@carbon fiber (Fe@NPC@CF)	−46.20	5.20	[141]
		Co@CNT@C	−53.50	8.02	[142]
		CF/restore oxidation graphene (RGO)/NiCo	−60.90	6.10	[144]
		RGO/Ni/C	−39.30	4.60	[145]
		Fe_3_O_4_@PC	−54.69	7.72	[146]
		TiO_2_@C/CF aerogels	−43.18	4.36	[149]
	Bamboo fiber	CoNi/carbonized bamboo fiber	−75.19	4.56	[154]
		PC/Fe	−43.20	4.72	[156]
		Carbon fiber aerogel@C/Co_3_O_4_	−43.50	7.84	[157]
		Graphene oxide/PANI/bamboo powder	−44.00	5.36	[161]
Other biomass materials	Laver	Ni@BPC	−35.73	6.37	[163]
	Kelp	Kelp porous carbon/Fe_3_O_4_	−75.02	4.83	[164]
	Rose	Rose-derived carbon materials/Co (RC/Co)	−47.89	4.08	[165]
	Pine cone	CeO_2_/PC	−56.04	5.28	[166]
	Coffee grounds	PC/Fe	−52.68	6.40	[167]
	Apple	Biocarbon/CoFe@C	−72.57	5.25	[168]
	Straw	Carbon black/Co@C	−53.99	6.00	[171]
		NiCo/straw-derived carbon	−27.00	4.40	[172]
		FeCl_3_/straw-derived carbon	−30.03	4.17	[173]

## Data Availability

Data are contained within the article.
